# Danger signal extracellular calcium initiates differentiation of monocytes into SPP1/osteopontin-producing macrophages

**DOI:** 10.1038/s41419-022-04507-3

**Published:** 2022-01-12

**Authors:** Supriya Murthy, Isabel Karkossa, Caroline Schmidt, Anne Hoffmann, Tobias Hagemann, Kathrin Rothe, Olga Seifert, Ulf Anderegg, Martin von Bergen, Kristin Schubert, Manuela Rossol

**Affiliations:** 1 Devision of Rheumatology, Department of Endocrinology, Nephrology, Rheumatology, Leipzig University, 04103 Leipzig, Germany; 2 Department of Molecular Systems Biology, Helmholtz Centre for Environmental Research GmbH, 04318 Leipzig, Germany; 3 Helmholtz Institute for Metabolic, Obesity and Vascular Research (HI-MAG) of the Helmholtz Zentrum München at the University of Leipzig and University Hospital Leipzig, 04103 Leipzig, Germany; 4 Department of Dermatology, Venereology and Allergology, Faculty of Medicine, Universität Leipzig, 04103 Leipzig, Germany; 5 Institute for Biochemistry, Faculty of Life Sciences, Leipzig University, 04318 Leipzig, Germany; 6 German Centre for Integrative Biodiversity Research (iDiv) Halle-Jena-Leipzig, 04103 Leipzig, Germany

**Keywords:** Rheumatoid arthritis, Innate immunity

## Abstract

The danger signal extracellular calcium is pathophysiologically increased in the synovial fluid of patients with rheumatoid arthritis (RA). Calcium activates the NLRP3-inflammasome via the calcium-sensing receptor in monocytes/macrophages primed by lipopolysaccharide, and this effect is mediated by the uptake of calciprotein particles (CPPs) formed out of calcium, phosphate, and fetuin-A. Aim of the study was to unravel the influence of calcium on monocytes when the priming signal is not present. Monocytes were isolated from the blood of healthy controls and RA patients. Macrophages were characterized using scRNA-seq, DNA microarray, and proteomics. Imaging flow cytometry was utilized to study intracellular events. Here we show that extracellular calcium and CPPs lead to the differentiation of monocytes into calcium-macrophages when the priming signal is absent. Additional growth factors are not needed, and differentiation is triggered by calcium-dependent CPP-uptake, lysosomal alkalization due to CPP overload, and TFEB- and STAT3-dependent increased transcription of the lysosomal gene network. Calcium-macrophages have a needle-like shape, are characterized by excessive, constitutive SPP1/osteopontin production and a strong pro-inflammatory cytokine response. Calcium-macrophages differentiated out of RA monocytes show a stronger manifestation of this phenotype, suggesting the differentiation process might lead to the pro-inflammatory macrophage response seen in the RA synovial membrane.

## Introduction

The danger signal extracellular calcium activates the NLRP3-inflammasome via the calcium-sensing receptor (CaSR) in monocytes/macrophages primed by a toll-like receptor-mediated signal like bacterial lipopolysaccharide (LPS) [1, 2]. Increased extracellular calcium concentrations are found near dying or activated cells [2, 3], at sites of chronic infections [4, 5], in the synovial fluid of patients with rheumatoid arthritis (RA) [6], in dialysis-related peritonitis [7], and in erosion sites beneath osteoclasts [8].

We have reported previously, that extracellular calcium alone is not sufficient to induce NLRP3 activation in monocytes, increased concentrations of phosphate are also required [6]. Calcium and phosphate ions and serum protein fetuin-A form 70–100 nm-sized calciprotein particles (CPP) to neutralize excess calcium and prevent calcification, and the three components are also liberated during bone resorption [8, 9]. CPPs are then taken up by monocytes/macrophages via macropinocytosis, and the process is enhanced by CaSR activation [6, 10, 11]. This leads to increased lysosomal activity, NLRP3-inflammasome activation and inflammasome-related cell death [6].

RA is an autoimmune disease characterized by synovial inflammation and bone erosions in joints. High numbers of inflammatory macrophages are found in the inflamed synovial tissue [12], which are probably derived from blood monocytes [13, 14]. We have reported previously that the synovial fluid of RA patients contains higher calcium ion concentrations and that monocytes of RA patients express more CaSR [6].

The aim of the study was to unravel the influence of calcium and CPPs on monocytes from healthy donors and RA patients when a priming signal like LPS is not present, and to dissect the subsequent intracellular events and functional consequences.

## Results

### Danger signal extracellular calcium and CPPs induce differentiation of monocytes into macrophage-like cells

**Fig. 1:**
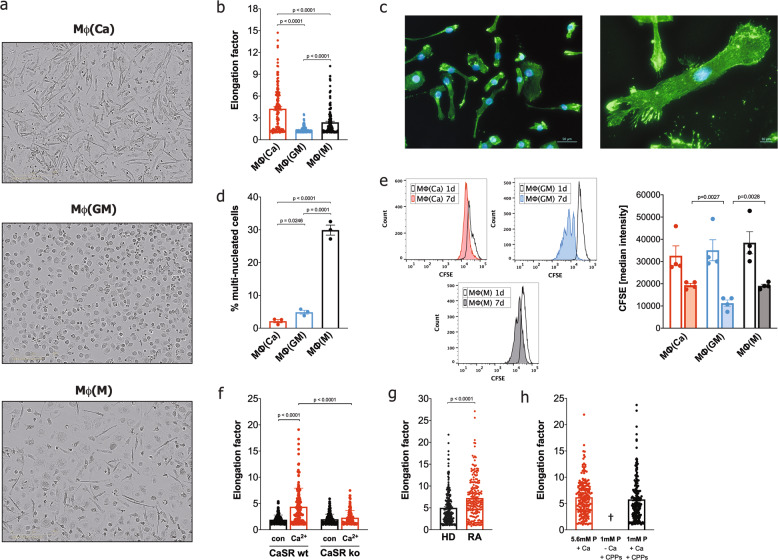
Calcium induces macrophage differentiation without additional growth factors. **a** Representative phase contrast images of calcium-macrophages (Mϕ(Ca)), GM-CSF-macrophages (Mϕ(GM)), and M-CSF-macrophages (Mϕ(M)) at 20x magnification (scale bar: 200 µm). **b** Quantification of cell elongation (length of the long axis divided by the length of the short axis). 138 Mϕ(Ca), 208 Mϕ(GM), and 148 Mϕ(M) of three different donors were analyzed. **c** Representative fluorescence images of (Mϕ(Ca) immunostained for filamentous actin (green) and Hoechst nuclear counterstain (blue). Scale bars: 50 µm (left), 10 µm (right). **d** Quantification of multi-nucleated cells. 235 Mϕ(Ca), 303 Mϕ(GM), and 167 Mϕ(M) of three different donors were analyzed. **e** Quantification of the proliferation of Mϕ(Ca), Mϕ(GM), and Mϕ(M). Shown are representative histograms of CFSE fluorescence and bar charts depicting the median CFSE fluorescence of macrophages from four different donors on day 1 (open bars) and day 7 (solid bars). **f** Quantification of cell elongation of differentiated control THP-1 cells (CaSR wt) or CaSR-deficient THP-1 cells (CaSR ko) incubated in the presence (Ca^2+^) or absence (con) of calcium. A total of 136 to 164 cells per condition from 4 different experiments were analyzed. **g** Quantification of cell elongation of Mϕ(Ca) from healthy donors (HD, *n* = 5, 230 cells) and RA patients (RA, *n* = 5, 211 cells). **h** Quantification of cell elongation of Mϕ(Ca) in RPMI1640/10%FBS containing 5.6 mM or 1 mM [P_i_] in the presence or absence of calcium (Ca) and calciprotein particles (CPPs). A total of 196 (5.6 mM [P_i_]) and 191 (1 mM [P_i_]) cells from 4 different donors were analyzed. **b**, **d-h** Bar charts show mean ± s.e.m. Statistical analysis was performed using two-tailed Mann–Whitney U test (**b**, **f**-**h**) or two-tailed *t* test (**d**, **e**).

We have previously shown that increased concentrations of extracellular calcium act as danger signal and lead to NLRP3-inflammasome activation and pyroptotic cell death in the presence of LPS [2, 6]. Here we show that the danger signal extracellular calcium induces macrophage differentiation and survival when LPS is absent. In the presence of increased extracellular calcium, monocytes differentiated into macrophage-like cells (calcium-macrophages), while the monocytes in the absence of additional survival factors and in only FCS-supplemented culture media, die after two days (Fig. 1a, Supplementary video 1, Fig. 1c). Calcium-macrophages have an extreme needle-like shape in contrast to both the round, “fried egg” shape of GM-CSF-macrophages and the elongated shape of M-CSF-macrophages (Fig. 1a). The quantification of cell elongation confirmed the visual observation of the extreme needle-like shape of calcium-macrophages (Fig. 1b). Almost all calcium-macrophages only contained one nucleus, whereas a substantial number of the M-CSF-macrophages contained two or more nuclei (Fig. 1d).

Monocytes without calcium or growth factors die within two days of cell culture. During calcium-induced differentiation of monocytes, substantial cell death was also observed during the first two days of differentiation. On day two of calcium-induced differentiation, 57.0% ± 5.3 viable cells were detected compared to 97.0% ± 0.8 in GM-CSF-macrophage cultures or 96.0% ± 0.3 in M-CSF-macrophage cultures. On day seven, the cell viability was > 97% in all macrophage cultures. Estimation of cell proliferation using CFSE revealed that calcium-macrophages as well as M-CSF-macrophages undergo one or no cell division during differentiation, whereas GM-CSF-macrophages divide one to four times (Fig. 1e). The yield of calcium-macrophages generated from human monocytes is lower than the yields of GM-CSF-macrophages and M-CSF-macrophages generated from monocytes after seven days (mean ± s.e.m, 27.6% ± 2.2 vs. 67.8% ± 5.8 *p* < 0.0001 and 52.8% ± 4.4, *p* < 0.0001, *n* = 10). Nevertheless, all monocyte-derived macrophage cultures reached high confluency after seven days, although the calcium-macrophage cultures had a lower cell density. The cell death during the initial two days of differentiation induced by calcium was only partially compensated for by cell proliferation, but instead the surviving cells seemed to grow in length to reach 100% confluency.

To explore the role of CaSR in the differentiation of calcium-macrophages, a CaSR-deficient monocytic THP-1 cell line was established using CRISPR-Cas9 technology (Supplementary Fig. 1). The addition of calcium to PMA-differentiated THP-1 cells led to the differentiation of elongated, needle-shaped calcium-macrophages in the control THP-1 cells, whereas the calcium-induced differentiation of CaSR-deficient THP-1 was abrogated, demonstrated by the complete absence of elongated macrophages (Fig. 1f).

We have shown previously that monocytes of RA patients have an increased expression of CaSR [6]. Accordingly, monocytes of RA patients differentiate to calcium-macrophages with a higher elongation factor than monocytes from healthy donors (Fig. 1g).

Increased concentrations of extracellular calcium ions lead to the formation of CPPs composed of calcium, phosphate, and fetuin-A, and these particles are taken up by monocytes by calcium-induced macropinocytosis [6]. To study the influence of CPPs on the differentiation of calcium-macrophages, CPPs were prepared and used to induce macrophage differentiation. As shown in Fig. 1h, CPPs induce the differentiation of calcium-macrophages when the uptake of CPPs by macropinocytosis was facilitated by the presence of calcium.

### Calcium-macrophages are different from GM-CSF-macrophages and M-CSF-macrophages

**Fig. 2:**
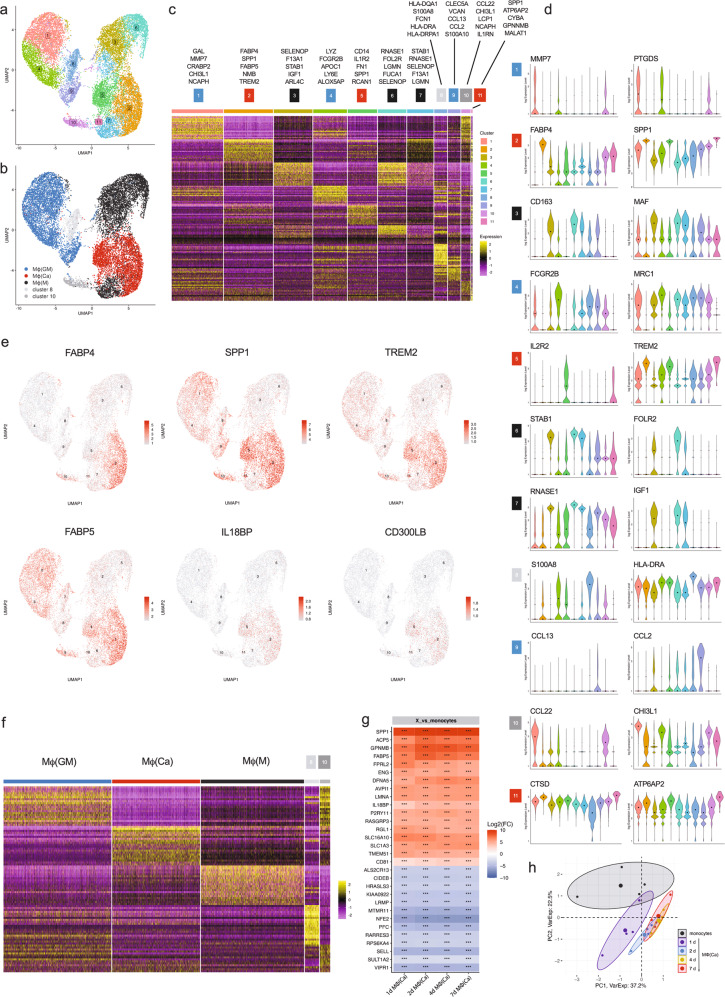
scRNA-seq defines a unique gene expression pattern of calcium-macrophages. **a** UMAP of 11 macrophage clusters identified by scRNA-seq analysis. **b** UMAP of the identified clusters with the detected macrophage population hashtag. **c** Heatmap of the top 20 DEGs per cluster. Top cluster markers and hashtag macrophage population assignment are provided. **d** Violin plots representing log-normalized expression values of cluster markers; medians marked by black dots and cluster identity by individual coloring. **e** Split UMAP and dot plots of relative changes in clusters. **f** Heatmap of the top 20 DEGs per macrophage population hashtag. **a–f** scRNA-seq of three tagged macrophage populations (7-day human monocyte-derived macrophages: calcium-macrophages, GM-CSF-macrophages, M-CSF-macrophages) pooled in one sample. **g** Top 30 genes significantly regulated in day 1 calcium-macrophages compared to monocytes. Calcium-macrophages were differentiated from monocytes of four different donors for the indicated times, and gene expression was analyzed using DNA microarray. Significance levels are marked with * *p* ≤ 0.05, ** *p* ≤ 0.01, *** *p* ≤ 0.001. **h** Principal component analysis of monocytes and day 1, day 2, day 4, and day 7 calcium-macrophages.

To determine the heterogeneity of calcium-macrophages in comparison to GM-CSF and M-CSF-macrophages, scRNA-seq was performed with resting, unstimulated macrophages. The three macrophage populations were labeled with different oligo-tagged antibodies, the cell populations were combined in one sample, and these tags were sequenced alongside the cellular transcriptome (CITE-seq, see Materials and Methods). In total, 16470 macrophages (4362 calcium-macrophages, 6588 GM-CSF-macrophages, 5520 M-CSF-macrophages) were analyzed after filtering. As shown in Fig. 2a, 11 distinct cell clusters were identified. Using the specific tags, clusters 2, 5, and 11 were assigned to calcium-macrophages (Fig. 2b, red), clusters 1, 4, and 9 to GM-CSF-macrophages (Fig. 2b, blue), and clusters 3, 6, and 7 to M-CSF-macrophages (Fig. 2b, black). Clusters 8 and 10 were not mapped to any of the three macrophage populations. Dimension reduction of the high dimensional gene expression space shows a clear separation of the macrophage populations suggesting that calcium-macrophages are distinct from GM-CSF-macrophages and M-CSF-macrophages (Fig. 2b).

A heatmap of the top-20 differentially expressed genes (DEG) per cluster, which are differentially expressed compared to all other clusters (Supplementary Table 1), and top cluster markers are shown in Fig. 2c, d. SPP1 (secreted phosphoprotein 1, osteopontin) was identified as top marker gene for all three calcium-macrophage clusters (Fig. 2c, d) and the expression of SPP1 was higher in calcium-macrophages than in control macrophages (Fig. 2d, e). Other important marker genes of calcium-macrophages are shown in Fig. 2d, e, KEGG pathways of expressed genes in calcium-macrophages are shown in Supplementary Fig. 2.

To compare the three macrophage populations, the top-20 DEGs of the labeled populations were plotted in a heatmap (Fig. 2f). This analysis revealed a distinct gene expression pattern in all three macrophages populations, suggesting that it is not possible to map calcium-macrophages to either GM-CSF-macrophages or M-CSF-macrophages and that at least in the resting state, calcium-macrophages are a unique macrophage population.

To analyze the gene expression patterns of calcium-macrophages during differentiation, DNA microarray analysis (n = 4) was performed. As shown in Fig. 2g, h, the most prominent changes in gene expression happen during the first two days after initiation of differentiation. The principal component analysis in Fig. 2h reflects the prominent change in gene expression on day-1 after initiation of differentiation, while calcium-macrophages on day-2 to day-7 are more similar. Top-enriched KEGG pathways based on the DEGs of day-1 calcium-macrophages compared to monocytes are shown in Supplementary Table 2.

**Fig. 3:**
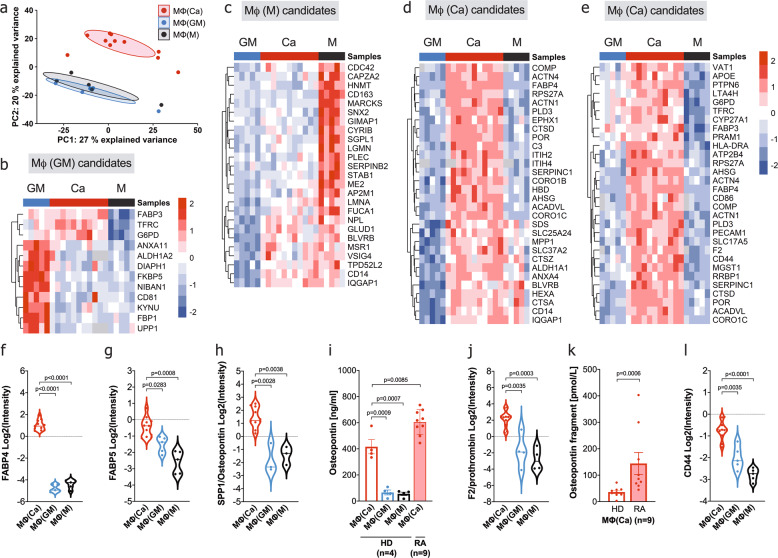
Calcium-macrophages have a distinct proteomic profile. **a-h**, **j**, **l** Proteomic analysis of cell lysates of calcium-macrophages (Ca, *n* = 11), GM-CSF-macrophages (GM, *n* = 5), and M-CSF-macrophages (M, *n* = 5) differentiated for 7 days. **i**, **k** Protein concentration in the supernatants of calcium-macrophages, GM-CSF-macrophages, and M-CSF-macrophages differentiated for 7 days. Bar charts show mean ± s.e.m. Statistical analysis was performed using two-tailed *t* test (**f**-**j**, **l**) or Mann–Whitney U-test (**k**). Significances in violin plots were adjusted for multiple testing as for the whole proteome. **a** Principal component analysis of the proteome of calcium-macrophages, GM-CSF-macrophages, and M-CSF-macrophages. **b** Significantly regulated proteins in GM-CSF-macrophages compared to M-CSF-macrophages. **c** Significantly regulated proteins in M-CSF-macrophages compared to GM-CSF-macrophages. **d** Top30 proteins significantly regulated in calcium-macrophages compared to GM-CSF-macrophages. **e** Top30 proteins significantly regulated in calcium-macrophages compared to M-CSF-macrophages. **f** Violin plot of FABP4 abundance. **g** Violin plot of FABP5 abundance. **h** Violin plot of SPP1/Osteopontin abundance. **i** Bar charts of SPP1/Osteopontin detected in the supernatants of macrophages. **j** Violin plot of F2/Prothrombin abundance. **k** Bar charts of cleaved Osteopontin detected in the supernatants of calcium-macrophages (HD healthy donors, RA RA patients, *n* = 9). **l** Violin plot of CD44 peptide abundance.

To determine the relation of resting calcium-macrophages to other macrophages on the protein level and to validate the scRNA-seq findings, proteomic analysis was performed. The proteomic signature of calcium-macrophages was also distinct from control macrophages (Fig. 3a), and this was confirmed based on the significant changes that were observable between the macrophages (Supplementary Fig. 3). Analysis of the significantly upregulated proteins of control macrophages (Fig. 3b, c) revealed that calcium-macrophages share only a few of these identified marker proteins. When the most significantly upregulated proteins of calcium-macrophages (Fig. 3d, e) were compared, a distinct proteomic profile of calcium-macrophages was visible. Top-regulated proteins included FABP4 (Fig. 3f), FABP5 (Fig. 3g), cathepsin D, and CD14 which were already identified using scRNA-seq. Calcium-macrophages differentiated out of monocytes from healthy donors and RA patients showed a comparable proteome signature (Supplementary Figs. 4 and 5). The calcium-macrophage proteome signature is not present in freshly isolated monocytes from healthy donors and RA patients (Supplementary Fig. 6).

SPP1/Osteopontin was found as a marker gene of calcium-macrophages using scRNA-seq and microarray analysis. In the proteomic analysis, SPP1/Osteopontin was also up-regulated in calcium-macrophages compared to control macrophages (Fig. 3h). SPP1/Osteopontin is an extracellular matrix protein in mineralized tissues and a cytokine in body fluids, which is secreted by macrophages [15]. Resting calcium-macrophages secreted SPP1/Osteopontin in high concentrations, and calcium-macrophages from RA patients produced SPP1/Osteopontin in even higher concentrations (Fig. 3i).

SPP1/Osteopontin can be modified by thrombin cleavage which exposes an epitope for integrin receptors [16]. Interestingly, F2/prothrombin is one of the top30-regulated proteins (Fig. 3e) and was strongly upregulated in calcium-macrophages (Fig. 3j). Accordingly, cleaved SPP1/Osteopontin was detected in the supernatants of calcium-macrophages and in increased concentrations in the supernatants of RA calcium-macrophages (Fig. 3k). Furthermore, CD44, a receptor for SPP1/Osteopontin [17], was one of the most significantly regulated proteins (Fig. 3e), and was upregulated in calcium-macrophages (Fig. 3l).

### Calcium-macrophages have a pro-inflammatory and migratory phenotype

**Fig. 4:**
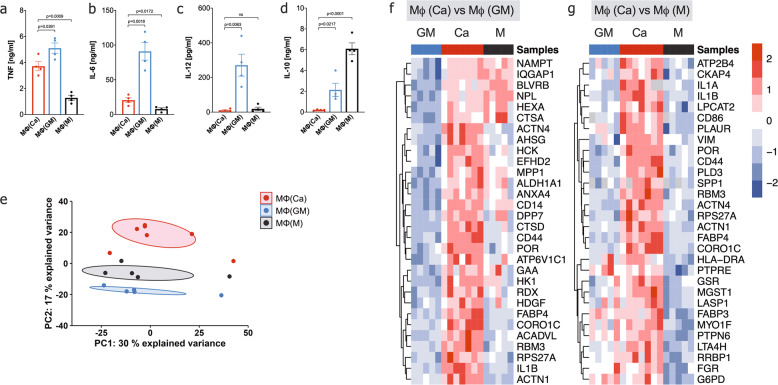
Activated calcium-macrophages are different from GM-CSF- and M-CSF-macrophages. **a-d** Bar charts of cytokines detected in the supernatants of macrophages stimulated with 10 ng/ml LPS for 24 h (*n* = 4). Bar charts show mean ± s.e.m. Statistical analysis was performed using two-tailed *t* test. **e** Principal component analysis of the proteome of calcium-macrophages (*n* = 7), GM-CSF-macrophages (*n* = 5), and M-CSF-macrophages (*n* = 5) stimulated with 10 ng/ml LPS for 24 h. **f** Top30 proteins significantly regulated in calcium-macrophages compared to GM-CSF-macrophages. **g** Top30 proteins significantly regulated in calcium-macrophages compared to M-CSF-macrophages.

To test whether calcium-macrophages are more similar to GM-CSF-macrophages or to M-CSF-macrophages after activation, macrophages were activated with LPS. LPS led to the secretion of the classical pro-inflammatory cytokines tumor necrosis factor (TNF), IL-6, and IL-12, whereas the anti-inflammatory cytokine IL-10 was barely detectable in the supernatants of calcium-macrophages (Fig. 4a–d).

Next, proteomic analysis of LPS-stimulated macrophages was performed. We found that the proteome signature of LPS-stimulated calcium-macrophages is distinct from control macrophages (Fig. 4e), but the lower numbers of significantly affected proteins obtained with LPS compared to the unstimulated macrophages (Supplementary Fig. 5) indicate that activation tempered the differences between the macrophage types. However, the comparison of the most significantly upregulated proteins of LPS-stimulated calcium-macrophages in comparison to control macrophages (Fig. 4f, g) revealed again a distinct protein signature, with the upregulation of pro-inflammatory cytokines IL-1α/IL-1β. LPS-activated calcium-macrophages differentiated out of monocytes from healthy donors and RA patients showed a comparable proteome signature (Supplementary Figs. 3 and 4).

We have previously shown that LPS together with the danger signal extracellular calcium activates the NLRP3 inflammasome, which leads to the secretion of IL-1β and IL-18 [2, 6].

**Fig. 5:**
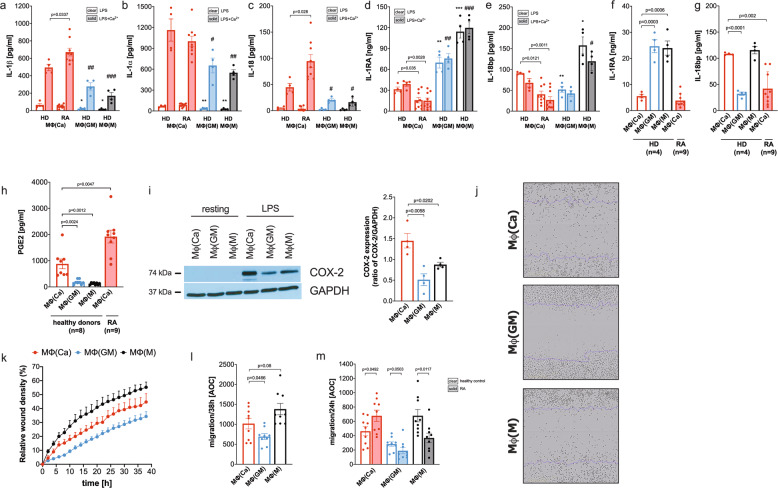
Calcium-macrophages have a pro-inflammatory and invasive phenotype. **a**–**g** Bar charts of cytokines detected in the supernatants of macrophages stimulated with 10 ng/ml LPS or LPS and 2.5 mM calcium for 24 h (**a**–**e**) or incubated without additional stimuli (**f**, **g**). Macrophages were differentiated from monocytes from healthy donors (HD, *n* = 4) or RA patients (RA, *n* = 9). **h** PGE2 detected in the supernatants of macrophages stimulated with 10 ng/ml LPS for 24 h. Macrophages were differentiated from monocytes from healthy donors (HD, *n* = 4) or RA patients (RA, *n* = 9). **i** COX-2 expression in resting macrophages or macrophages stimulated with 10 ng/ml LPS for 24 h (*n* = 4). Shown is one representative western blot and quantification of COX-2 expression. **j–m** Scratch assay of macrophage monolayers (*n* = 9). **j** Representative phase contrast images taken with IncuCyte. Purple line indicates the mask for initial scratch width. Magnification 10x. **k** Change of relative wound density during 38 h after scratch. **l** Area under the curve (AOC) of relative wound density. **m** Area under the curve (AOC) of relative wound density of macrophages from healthy donors (*n* = 9) and RA patients (*n* = 9). **a–i**, **l**, **m** Bar charts show mean ± s.e.m. Statistical analysis was performed using two-tailed *t* test. Significance levels are given or marked with * *p* ≤ 0.05, ** *p* ≤ 0.01, *** *p* ≤ 0.001 (comparisons between LPS-stimulated macrophages and calcium-macrophages) or # *p* ≤ 0.05, ## *p* ≤ 0.01, ### *p* ≤ 0.001 (comparisons between LPS/calcium-stimulated macrophages and calcium-macrophages).

Here we show that activated calcium-macrophages produce higher amounts of pro-inflammatory IL-1β, IL-1α, and IL-18 than control macrophages (Fig. 5a–c), and in contrast, the anti-inflammatory IL-1-receptor-antagonist (IL-1RA) and IL-18bp is produced in lower amounts by calcium-macrophages (Fig. 5d, e). RA calcium-macrophages responded with an even higher pro-inflammatory and a lower anti-inflammatory cytokine production compared to healthy donors (Fig. 5a–e). Both anti-inflammatory cytokines were also secreted by resting calcium-macrophages (Fig. 5f, g).

DNA microarray analysis of LPS-stimulated calcium-macrophages revealed a distinct gene profile as well (Supplementary Fig. 7), most notably with the upregulation of Prostaglandin E synthase (PTGES). Prostaglandin E2 (PGE2) is a derivate of arachidonic acid, produced by constitutively active cyclooxygenase COX1, inducible COX2, and PTGES [18]. Here we show that calcium-macrophages produce higher amounts of PGE2 than control macrophages (Fig. 5h), and express higher amounts of COX2 (Fig. 5i). PGE2 is found in high concentrations in the synovial fluid of RA patients [19]. Calcium-macrophages from RA patients produce high amounts of PGE2, exceeding the already high levels produced by calcium-macrophages from healthy donors (Fig. 5h).

To check the migratory potential of resting calcium-macrophages, a scratch assay was performed. As shown in Fig. 5j-l, M-CSF-macrophages and calcium-macrophages exhibit the highest migratory potential (Fig. 5k, l, m). RA calcium-macrophages have an even higher migratory potential, whereas the migration of control macrophages is lower in RA patients compared to healthy controls (Fig. 5m).

### Calciprotein particles are not degraded in the lysosomes

**Fig. 6:**
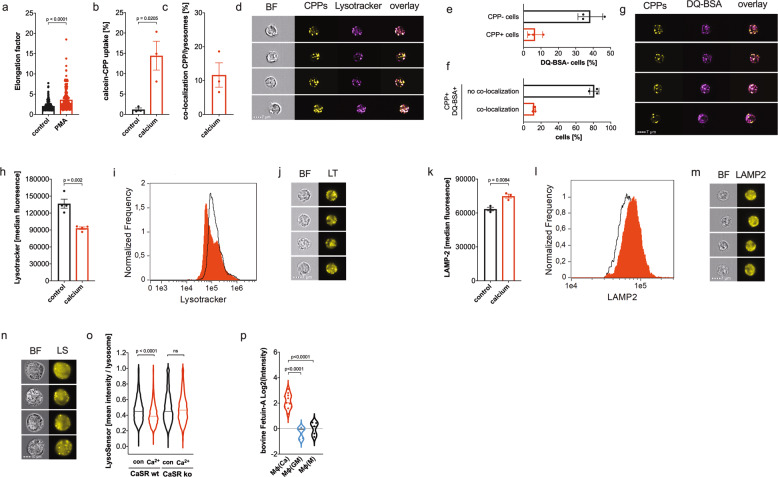
Calciprotein particles are not degraded in the lysosomes. **a** Quantification of cell elongation of calcium-differentiated CaSR-deficient THP-1 cells in the presence (PMA) or absence (control) of PMA. A total of 200 cells per condition from 4 different experiments were analyzed. Bar chart show mean ± s.e.m. Statistical analysis was performed using two-tailed *t* test. **a**–**d** Calcium-dependent uptake of calcein-labeled CPPs (**b**) and co-localization of calcein-CPPs with lysosomes (**c**) stained with lysotracker in monocytes stimulated with and without 2.5 mM CaCl_2_ for 4 h. Analysis was performed using ImageStreamX Mark II and 5000–10,000 monocytes were imaged from 3 different donors. Representative images showing brightfield (BF), calcein-CPPs, lysotracker, and an overlay of CPP and lysotracker staining (**d**). Bar charts show mean ± s.e.m. Statistical analysis was performed using two-tailed *t* test. **e-g** Uptake of DQ-BSA in calcein-CPP + and CPP- negative monocytes (**e**) and co-localization of calcein-CPPs with DQ-BSA in calcein-CPP + monocytes (**f**) stimulated with 2.5 mM CaCl_2_ for 4 h. Analysis was performed using ImageStreamX Mark II and 5000–10,000 monocytes were imaged from 3 different donors. Representative images showing calcein-CPPs, DQ-BSA, and an overlay of CPP and DQ-BSA staining (**g**). Bar chart show mean ± s.e.m. Statistical analysis was performed using two-tailed *t* test. **h–j** Analysis of lysosomes using lysotracker in monocytes stimulated with and without 2.5 mM CaCl_2_ for 3 h. Analysis was performed using ImageStreamX Mark II and 5000–10,000 monocytes were imaged from 4 different donors. Bar chart show mean of median fluorescence ± s.e.m. Statistical analysis was performed using two-tailed *t* test (**h**). Representative histogram of lysotracker fluorescence in control monocytes (white) and calcium-treated monocytes (red) (**i**), and representative images showing brightfield (BF) and lysotracker (LT) (**j**). **k–m** Analysis of lysosomes using LAMP2 in monocytes stimulated with and without 2.5 mM CaCl_2_ for 3 h. Analysis was performed using ImageStreamX Mark II and 5000–10,000 monocytes were imaged from 4 different donors. Bar chart show mean of median fluorescence ± s.e.m. Statistical analysis was performed using two-tailed *t* test (**k**). Representative histogram of LAMP2 fluorescence in control monocytes (white) and calcium-treated monocytes (red) (**l**), and representative images showing brightfield (BF) and LAMP2 (**m**). **n**, **o** LysoSensor staining of differentiated control THP-1 cells (CaSR wt) or CaSR-deficient THP-1 cells (CaSR ko) incubated in the presence (Ca^2+^) or absence (con) of calcium for 3 h. Analysis was performed using ImageStreamX Mark II and 5000–10,000 monocytes were imaged from 3 different experiments. Representative images showing brightfield (BF) and LysoSensor (LS) (**n**). Violin plot show median of the mean intensity of lysosomes. A total of 168 cells and 6900–8500 lysosomes were analyzed per condition. Statistical analysis was performed using Mann–Whitney U-test (**o**). **p** Violin plot of bovine Fetuin-A abundance in the proteome of calcium-macrophages (*n* = 11), GM-CSF-macrophages (*n* = 5), and M-CSF-macrophages (*n* = 5).

Monocytes take up CPPs by calcium-induced, CaSR-dependent macropinocytosis [6]. CaSR-deficient THP-1 cells are not able to macropinocytose CPPs because the macropinocytosis inducer calcium is not detected by the cells. PMA is a well-known inducer of macropinocytosis [20], and when macropinocytosis of CPPs was induced by PMA in CaSR-deficient THP-1 cells, the elongated calcium-macrophages were detected in contrast to the CaSR-deficient THP-1 cells without PMA (Fig. 6a), suggesting that the CaSR-deficiency is rescued by macropinocytosis stimulation.

Macropinosomes fuse with lysosomes to initiate degradation of macropinosome content [21]. We used lysotracker to stain the lysosomes, and co-localization of calcein-labeled CPPs with lysosomes was detected in only a minor part of the CPP-positive monocytes (Fig. 6b–d). To analyze the degradation of proteins in monocytic lysosomes, DQ-BSA was used. DQ-BSA is a self-quenched fluorogenic protease substrate that gets de-quenched when its degraded in the endo-lysosomes [22]. As shown in Fig. 6e–g, only a few CPP+ monocytes were also positive for DQ-BSA, whereas a substantial part of the CPP-negative monocytes contained fluorescent DQ-BSA. When the co-localization of calcein-labeled CPPs and DQ-BSA was analyzed in CPP+ monocytes, most of the DQ-BSA was found not to be co-localized with CPPs (Fig. 6f).

It was reported recently that CPPs increase the pH of the fused endosomes/lysosomes and that pH-sensitive dyes like lysotracker are not suitable to detect lysosomes under such circumstances [23]. And indeed, lysotracker fluorescence was reduced (Fig. 6h–j), whereas pH-independent LAMP2 staining of lysosomes was increased in calcium-stimulated monocytes (Fig. 6k–m), suggesting that calcium led to an increased pH of fused endosomes/lysosomes and an increased lysosome content. To analyze the pH of the lysosomes in calcium-macrophages, the LysoSensor dye was used. LysoSensor becomes more fluorescent in acidic environments. And as expected, the mean fluorescence intensity of 6900–8500 analyzed lysosomes was decreased in calcium-stimulated THP-1 cells, whereas no difference was observed in CaSR-deficient THP-1 cells (Fig. 6n, o).

The absence of DQ-BSA degradation when CPPs are present and the increased pH in fused endosomes/lysosomes of calcium-stimulated monocytes point to a failure to degrade CPPs and the contained fetuin-A, resulting in lysosomal stress. If fetuin-A is indeed not degraded after macropinocytosis, it should be still present in macrophages differentiated for seven days. Thus, the proteome was searched for unique human and bovine fetuin-A peptides and we found that calcium-macrophages contained more bovine fetuin-A than control macrophages (Fig. 6p).

### Disruption of lysosomal homeostasis induces TFEB and STAT3 transcription factors

It is known that lysosomal homeostasis and the mechanistic target of rapamycin (mTOR) pathway are linked, and when lysosome function is normal, the transcription factor EB (TFEB) is phosphorylated in an mTORC1-dependent manner and retained in the cytosol [24, 25]. When lysosomal function is impaired, mTORC1 is inactivated, and unphoshorylated TFEB translocates into the nucleus and initiates the expression of lysosomal proteins [24, 25].

**Fig. 7:**
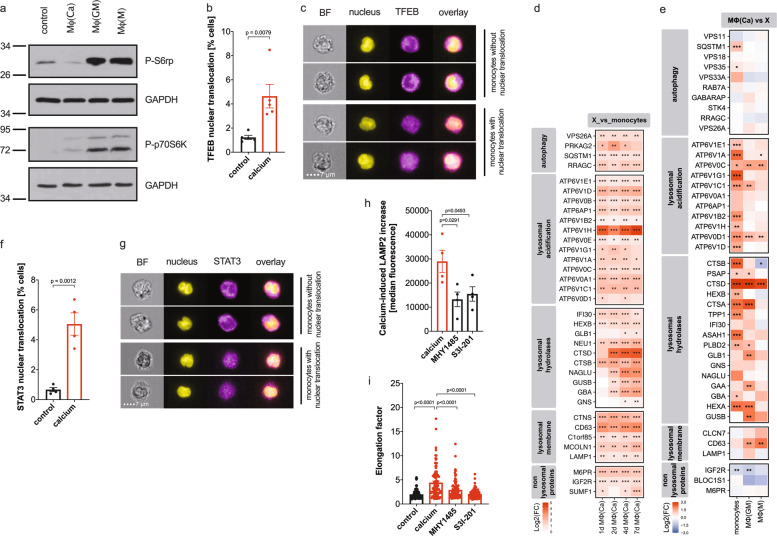
Lysosomal stress induces TFEB and STAT3 transcription factors. **a** Expression of phosphorylated S6rp (P-S6rp) and p70S6K (P-p70S6K) with respective GAPDH loading controls in monocytes differentiated for 1 day. Shown is one representative western blot. **b**, **c** Calcium-dependent nuclear translocation of TFEB in monocytes stimulated with and without 2.5 mM CaCl_2_ for 1 day (**b**). Analysis was performed using ImageStreamX Mark II and 5000–10,000 monocytes were imaged from 5 different donors. Representative images showing brightfield (BF), nucleus, TFEB, and an overlay of nucleus and TFEB staining (**c**). Bar charts show mean ± s.e.m. Statistical analysis was performed using Mann–Whitney U-test. **d** TFEB target genes significantly regulated in day 1, 2, 4, or 7 calcium-macrophages compared to monocytes. Calcium-macrophages were differentiated from monocytes of four different donors for the indicated times, and gene expression was analyzed using DNA microarray. Significant changes of calcium-macrophages compared to the other cells are indicated with asterisks (adjusted *p* value * ≤ 0.05, ** ≤ 0.01, *** ≤ 0.001). **e** Proteomic analysis of TFEB target proteins in cell lysates of fresh monocytes (*n* = 5), calcium-macrophages (*n* = 11), GM-CSF-macrophages (*n* = 5), and M-CSF-macrophages (*n* = 5) differentiated for 7 days. Significant changes of calcium-macrophages compared to the other cells are indicated with asterisks (adjusted *p* value *≤ 0.05, **≤ 0.01, ***≤ 0.001). **f**, **g** Calcium-dependent nuclear translocation of STAT3 in monocytes stimulated with and without 2.5 mM CaCl_2_ for 1 day (**f**). Analysis was performed using ImageStreamX Mark II and 5000–10,000 monocytes were imaged from 4 different donors. Representative images showing brightfield (BF), nucleus, STAT3, and an overlay of nucleus and STAT3 staining (**g**). Bar charts show mean ± s.e.m. Statistical analysis was performed using Mann–Whitney U-test. **h** Analysis of lysosomes using LAMP2 in monocytes stimulated with 2.5 mM CaCl_2_ for 3 h in the presence of 5 µM mTOR activator MHY1485 or 25 µM STAT3 inhibitor S3I-201. Analysis was performed using ImageStreamX Mark II and 5000–10,000 monocytes were imaged from 4 different donors. Bar charts show mean of median fluorescence ± s.e.m. Statistical analysis was performed using two-tailed *t* test. **i** Quantification of cell elongation of differentiated THP-1 cells (control) or calcium-differentiated THP-1 cells in the presence of DMSO (calcium), mTOR activator MHY1485 or STAT3 inhibitor S3I-201. A total of 200 cells per condition from 4 different experiments were analyzed. Bar chart show mean ± s.e.m. Statistical analysis was performed using two-tailed *t* test.

Here we found that the phosphorylation/activation of the downstream targets of mTORC1, S6 ribosomal protein (S6rp) and the ribosomal protein S6 kinase (p70S6K), are strongly reduced in calcium-macrophages compared to control macrophages (Fig. 7a, Supplementary Fig. 8), suggesting that mTORC1 is inactivated in calcium-stimulated monocytes/macrophages. Accordingly, the translocation of TFEB into the nucleus was increased in calcium-stimulated monocytes (Fig. 7b, c). Analysis of TFEB target genes [26] revealed a strong upregulation of the CLEAR (Coordinated Lysosomal Expression and Regulation) gene network during differentiation of calcium-macrophages (Fig. 7d), and this upregulation was also apparent in the proteome of calcium-macrophages differentiated for seven days (Fig. 7e).

The STAT3-signaling pathway is also important for regulating lysosomal homeostasis [27]. Substrate overload promotes the STAT3-dependent transcription of lysosomal proteins including Cathepsins B/D [27], which were found to upregulated among the TFEB target genes (Fig. 7d, e). Analysis of transcription factor targets using the TRRUST database [28] revealed that STAT3 is among the important transcription factors when DEGs of calcium-macrophages compared to monocytes (DNA microarray, see Fig. 2g) were analyzed (Supplementary top-20 differentially expressed genes (DEG) per cluster). The identified STAT3 target genes and their regulation during differentiation of calcium-macrophages are shown in Supplementary Fig. 9, and a part of the identified targets were also detectable in the proteome (Supplementary Fig. 10). Analysis of intracellular localization of STAT3 using ImageStream revealed that calcium-induced nuclear translocalization of STAT3 (Fig. 7f, g).

To test whether TFEB or STAT3 are involved in the calcium-induced increase in lysosomes, the mTOR activator MHY1485 (to facilitate mTORC1-dependent TFEB phosphorylation and cytosol retention) and the STAT3 inhibitor S3I-201 were used. Both led to an inhibition of the calcium-induced increase of LAMP2-fluorescence (Fig. 7h), suggesting that transcription factors TFEB and STAT3 are both involved in lysosomal protein expression and biogenesis. In the THP-1 model of calcium-macrophage differentiation, inhibition of TFEB nuclear translocation by mTOR activator MHY1485 led to a partial inhibition, and STAT3 inhibition by S3I-201 completely diminished calcium-macrophage differentiation (Fig. 7i).

## Discussion

We demonstrated that an increased concentration of extracellular calcium leads to the formation and uptake of CPPs and to the subsequent differentiation of monocytes into macrophages, and most strikingly, additional growth factors like GM-CSF or M-CSF are not needed for this process. Calcium-macrophages have a distinct morphological phenotype, they differentiate into needle-like cells. And although the M1/M2 model is outdated [29], we detected more pro-inflammatory cytokines and less anti-inflammatory IL-10 in GM-CSF-macrophages than in M-CSF-macrophages, resembling “pro-inflammatory M1” macrophages and “healing M2” macrophages, respectively. It was not possible to strictly assign calcium-macrophages to M1- or M2-macrophages, in contrast, they show a unique phenotype compared to control macrophages in the scRNA-seq, DNA microarray, proteomics, and functional analysis. This phenotype is characterized by the needle-like shape, an excessive constitutive SPP1/osteopontin production, a strong pro-inflammatory cytokine production, the absence of anti-inflammatory mediator secretion, and the pronounced migratory potential. Calcium-macrophages differentiated out of monocytes from RA patients show an overall stronger manifestation of this phenotype.

One of the most notable characteristics of calcium-macrophages is the excessive constitutive secretion of SPP1/osteopontin. SPP1/osteopontin is an extracellular matrix protein and pro-inflammatory cytokine, and is involved in many physiological and pathophysiological processes [30]. It was found to be expressed in higher levels in arthritic joints compared to nonarthritic joints in mice, SPP1/osteopontin-deficiency protected against joint destruction in collagen-antibody-induced arthritis, and increased expression was also observed in the synovial tissue of RA patients [31–34], but other studies found no influence of SPP1/osteopontin on murine arthritis models [35, 36]. Recently, SPP1/osteopontin-positive synovial macrophages were identified by Alivernini et al. using scRNA-seq in the synovial tissue of RA patients [37]. The SPP1-positive macrophage cluster was abundant in active RA, scarce in health and remission, correlated positively with disease activity, and contained high levels of cytoskeletal proteins and integrins suggesting a migratory phenotype [37].

SPP1/osteopontin can be modified by thrombin cleavage which exposes an epitope for integrin receptors [16], and prothrombin expression was found to be up-regulated in calcium-macrophages. The cleaved SPP1/osteopontin was detectable in the supernatants of calcium-macrophages, and in increased concentration in the supernatants of RA calcium-macrophages. It has been reported that thrombin-cleaved SPP1/osteopontin is present in the synovial fluid of RA patients [38], and that an antibody raised against the cryptic epitope of SPP1/osteopontin inhibits several features of arthritis in mice [16]. SPP1/osteopontin also induced a strong pro-inflammatory activation of monocytes, further aggravating inflammation [39].

The role of SPP1/osteopontin in the pathogenesis of RA remains controversial, however, the key pathogenic macrophages have a SPP1/osteopontin signature [37], and the here described SPP1/osteopontin-producing calcium-macrophages seem to resemble those macrophages.

In RA, monocytes are recruited continuously to the synovial tissue of the joint and differentiate into pro-inflammatory macrophages [13, 40], and a decrease in synovial macrophage numbers is associated with clinical improvement after treatment [41]. We have previously shown that the concentration of calcium ions is increased in the synovial fluid of RA patients [6]. Extracellular calcium is known to function as a chemokinetic agent [42], the local accumulation of calcium might facilitate monocyte recruitment into the joint and the differentiation of monocytes into pro-inflammatory calcium-macrophages, characterized by excessive SPP1/osteopontin secretion. In addition, we and others described previously that monocytes of RA patients express more CaSR [6, 43], which further promotes influx of monocytes into the synovial tissue. The increased CaSR expression of RA monocytes also leads to an increased macropinocytotic uptake of CPPs [6] promoting the calcium/CPP-induced differentiation of monocytes into calcium-macrophages.

We have previously reported that increased extracellular calcium leads to the formation of CPPs in the presence of phosphate and fetuin-A [6]. The differentiation of monocytes into calcium-macrophages also depends on the formation and uptake of CPPs. Recently it was reported that the uptake of CPPs leads to an increase of lysosomal pH [23], and we were able to confirm this. The disruption of lysosomal homeostasis after macropinocytotic uptake of CPPs in monocytes led to mTORC1 inactivation, and to the translocation of TFEB and STAT3 into the nucleus. mTORC1 co-localizes with TFEB on the lysosomal membrane when nutrients are present, and TFEB is phosphorylated by mTORC1 and retained in the cytosol [44]. When endosomal CPPs localize to the lysosomes, they increase the pH of the fused endosome/lysosome, and this increase is most likely mediated by the solubilization of CPPs and the increase of free calcium and phosphate ions in the lysosome [23]. The alkalization of the fused endosome/lysosome leads to a decrease of lysosomal hydrolase activity, to the accumulation of lysosomal storage material, inactivation of mTORC1, and TFEB nuclear translocation [23, 45]. TFEB is the master regulator for the CLEAR (Coordinated Lysosomal Expression and Regulation) gene network, resulting in lysosomal biogenesis [26, 46]. Calcium-macrophages showed a strong upregulation of the CLEAR gene network already one day after initiation of differentiation, and more lysosomal marker LAMP2 expression, suggesting an increase in lysosomal biogenesis in response to substrate overload. The STAT3 signaling pathway is also involved in regulating lysosomal homeostasis [27], and calcium-macrophages showed a strong upregulation of STAT3 target genes. STAT3/TFEB target genes are still up-regulated in differentiated calcium-macrophages, demonstrating a role of both transcription factors in the differentiation process.

In summary, increased concentrations of extracellular calcium and CPPs lead to the differentiation of monocytes into pro-inflammatory calcium-macrophages. Calcium-macrophages differentiated out of monocytes from RA patients show an overall stronger manifestation of this phenotype, implying that this differentiation process might lead to the pro-inflammatory macrophage response seen in the synovial membrane of RA patients.

## Methods

### Healthy controls and patients with rheumatoid arthritis

30 patients with RA according to the criteria of the American College of Rheumatology were included into the study. Patient characteristics are shown in Supplementary Table 4. Sex- and age-matched control subjects were recruited among healthy blood donors for experiments with comparisons between monocytes from healthy donors and RA patients.

### Monocyte isolation and macrophage differentiation

Peripheral blood mononuclear cells (PBMCs) were isolated by density gradient centrifugation using Ficoll-Paque (GE healthcare). PBMCs were washed with cold PBS containing 0.3 mM ETDA and monocytes were isolated by negative selection using human monocyte isolation kit II (Miltenyi Biotech) according to the manufacturer’s instructions.

Freshly isolated monocytes were differentiated into calcium-macrophages, GM-CSF-macrophages, and M-CSF-macrophages by incubating 5 × 10^5^/ml monocytes with differentiation media for seven days at 37 °C and 5% CO_2_. Calcium-macrophages were differentiated in RPMI1640 cell culture medium (Gibco, Life Technologies) supplemented with 10% FCS (Gibco, Life Technologies), 1% penicillin–streptomycin (Invivogen), and 2.5 mM Ca^2+^ (Sigma). GM-CSF and M-CSF macrophages were differentiated by culturing monocytes in RPMI1640 cell culture medium (Gibco, Life Technologies) with 10% FCS (Gibco, Life Technologies), 1% penicillin–streptomycin, 50 mM 2-Mercaptoethanol (Gibco, Life Technologies), 1 mM sodium pyruvate (Gibco, Life Technologies), 1 mg/ml NaHCO_3_ (Roth), 0.1% non-essential amino acids (Gibco, Life Technologies), 0.4% MEM vitamins (Gibco, Life Technologies), and 50 ng/ml animal-free recombinant human GM-CSF (Peprotech) or 50 ng/ml animal-free recombinant human M-CSF (Peprotech) for seven days.

Differentiated macrophages were either left unstimulated or stimulated with 10 ng/ml LPS (Invitrogen) for 1 day for further experiments. Adherent macrophages were harvested, and cell death was <3%, evaluated using trypan blue.

### Generation of a CRISPR/Cas9-mediated CaSR-knockout THP-1 cell line

THP-1 cells (DSMZ-German Collection of Microorganisms and Cell Cultures GmbH) were transduced with inducible lentiviral Cas9 particles (Dharmacon, Edit-R Inducible Lentiviral hEF1α-Blast-Cas9 Nuclease Particles). Due to the use of inducible Cas9, tetracyclin-free FBS was used for all experiments. 3 × 10^5^ THP-1 cells were seeded in 600 µl RPMI/1% FBS in a 48-well plate and cells were transduced at MOI 4. After 16 h, medium was removed and 500 μl RPMI 1640 with 10% FCS and 1% penicillin/streptomycin was added. Cells were selected 72 h after transduction with 10 μg/ml blasticidin for one week.

Subsequently, THP-1 inducible Cas9 cells were transduced with lentivirus containing the sgRNA against CaSR (Dharmacon, Edit-R Custom Lentiviral sgRNA, DNA target sequence 5ʹ-TCCCACCACAGCAATCGTAG-3ʹ) or a non-targeting control sequence (Edit-R Lentiviral mCMV-Puro non-targeting sgRNA particles) as described above. In total, 72 h after transduction, cells were selected by adding 0.8 µg/ml puromycin and 10 µg/ml blasticidin. Cas9 expression was induced by addition of 100 ng/ml doxycycline for 3 days and was detected by Western blot (Cell Signaling, Cas9 antibody 7A9-3A3, Cat. no. 14697 S).

A p24 ELISA (Sino Biologicals, Cat. no. KIT11695) was used to detect the absence of virus in the cell culture supernatant and was performed according to the manufacturer’s instructions.

Single cell clones were plated on BD FACS Aria III Cell Sorter (Core Unit Fluorescence Technologies, Leipzig University). For this purpose, single cells were added into 75 µl each of fresh cell culture medium (RPMI 1640 with 10% FCS and 1% Pen/Strep) and conditioned cell culture medium from on-going cultures, filtered through a 0.2 µm filter, into a 96-well round-bottom plate. Plates were sealed with parafilm and incubated at 37 °C for 3 weeks.

The deletions in CaSR gene were identified using PCR-based Guide-it Mutation Detection Kit (TaKaRa) (primers: forward 5ʹ- TCTTCCCAACTTGACGCTGG -3ʹ and reverse 5ʹ- CAGATTTGCCACTGCCGTG -3ʹ). For further experiments, a clone with a successful deletion (CaSR_1 C6) and a control clone without deletion (Ctrl171 A4) were chosen.

PCR-based sequencing was performed to verify the base pair deletions within the CaSR gene. The PCR products (primers: forward 5ʹ- GCATGCCATGAAGCCAGAGA -3ʹ and reverse 5ʹ- TCTTTTGTGCCAGAGATGGGA -3ʹ) were sequenced in the Core Unit DNA Technologies (Leipzig University). In CaSR_1 C6 cells no wildtyp gene could be detected. All sequences showed a 14-bp or 44-bp deletion, which was not present in Ctrl171 A4 cells. The cells tested negative for mycoplasma.

### scRNA-seq

Calcium-macrophages, GM-CSF-macrophages, and M-CSF-macrophages from one healthy donor were pooled together into one sample to perform 10xGenomics single cell RNA sequencing (scRNA-seq). To label the macrophage populations with oligo-tagged antibodies, 2 million cells of each macrophage population were blocked using 1:10 dilution of Human TruStain FcX™ Fc Blocking reagent (Bio Legend) and tagged with 1 µg of TotalSeq hashtag (Bio Legend) in Cell Staining Buffer (Bio Legend, 420201). TotalSeq™-B 0251 anti-human Hashtag (394631) was used for GM-CSF-macrophages, TotalSeq™-B 0252 anti-human Hashtag (394633) for M-CSF-macrophages, and TotalSeq™-B 0253 anti-human Hashtag (394635) for calcium-macrophages. scRNA-seq was performed in the Dresden-concept Genome Center, Center for Regenerative Therapies Dresden (CRTD), Cluster of Exellence at TU Dresden, Germany. In brief, cells were mixed with the reverse transcription mix before loading them in a Chromium Single Cell G Chip on the 10X Genomics Chromium device and processed further following the guidelines of the 10x Genomics user manual (v3.1). The droplets were directly subjected to reverse transcription, the emulsion was broken and cDNA was purified using silane beads. After amplification of cDNA with 11 cycles using primers to enrich cDNA as well as Totalseq-B hashtag, it underwent a cleanup, including a fractionation of small fragments (up to 400 bp) to enrich the hashtag sequences and larger fragments (>400 bp) to separate cDNA fragments. After quality check and quantification, the 10X Genomics single cell RNA-seq library preparation involving fragmentation, dA-Tailing, adapter ligation and a 12 cycles indexing PCR was performed based on the manufacturer’s protocol.

In parallel, the hashtag library was prepared by a 12-cycles index PCR. After quantification, both libraries were sequenced on an Illumina Novaseq 6000 in 100 bp paired-end mode, thus generating ~ 400 mio. fragments for the transcript library and 45 mio. fragments for the hashtag library. The raw sequencing data was then processed with the ‘count’ command of the Cell Ranger software (v5.0.1) provided by 10X Genomics with the option ‘--expect-cells’ set to 10,000 (all other options were used as per default).

Raw scRNA-seq reads were processed with the 10xGenomics pipeline which generated gene expression matrices. Prior to dimension reduction and clustering, apoptosis-related genes and genes which show expression in less than ten barcodes were removed. Droplet defining barcodes were filtered based on gene expression counts as outlined in Luecken and Theis [47]. To remove empty and low quality droplets, e.g. containing dying cells, barcodes were removed from the analysis if they met one of the following conditions: genes per barcode <500 or >6000, unique molecular identifiers (UMI) per barcode < 500, ratio of number of genes per number of UMI >0.8 and a ratio of mitochondrial genes compared to all genes >0.25 (Supplementary Fig. 11). These thresholds were defined based on the respective distributions of each parameter. Afterwards, normalization, transformation, dimension reduction and clustering of the filtered gene expression matrix were performed according to the standard workflow with the R package Seurat (v4.0.1) [48]. Clusters were assigned to macrophage populations based on the oligo-tagged antibody labeling signals (Supplementary Fig. 12). DEGs were identified for each cluster and macrophage population compared to all other clusters and all macrophage populations, respectively. For this purpose, the Wilcoxon test was applied for all genes with a presence fraction in either group >0.25 and a log fold change > 0.25 (adjusted *p* values < 0.01) using the FindAllMarkers function from the Seurat package.

Cluster 7 contained 7 mitochondrial encoded genes among the strongest DEGs (see Supplementary Table 1), which were not considered cluster markers and not shown in Fig. 2.

Subsequently, the set of DEGs for calcium-macrophages was subject to pathway enrichment analysis with the R package enrichR (v. 3.0) [49] to identify significantly enriched pathways (*p* < 0.05) according to the resources Kyoto Encyclopedia of Genes and Genomes (KEGG) [50]. scRNA-seq data were deposited in the NCBI GEO database under accession number GSE180113.

### DNA microarray

RNA from monocytes-derived macrophages (*n* = 4) was isolated using Trizol (Invitrogen) and further precipitated using ammonium acetate (Sigma). DNA microarray was performed in the DNA core unit facility, University of Leipzig. 250-500 ng of RNA was used for cDNA preparation and in vitro transcription (Epicentre Reagents, Illumina).

Whole-genome gene expression direct hybridization assay was performed with BeadChip array HumanHT-12_v4_BeadChip (Illumina Hybridiation Oven Model 5521, iSCan and AutoLoader2). The data were background corrected and quantile normalized using the neqc function from the limma R package (v3.46.0) [51]. Based on the annotation, probes with bad or no matched quality were excluded from further analysis. Probes that are expressed in at least three arrays according to a detection p-value of 5 were filtered out. Quality control was performed with the limma and arrayQualityMetrics (v 3.46.0) R packages [52]. The DEGs for the comparisons 1-day calcium-macrophages versus monocytes, and LPS-stimulated calcium-macrophages versus GM-CSF-macrophages were screened using the linear models for microarray data (LIMMA) method [51]. To increase the signal-to-noise ratios, array weights were fitted in the linear model. As the study includes multiple cell types from the same donor which are consequently not independent, we estimated the within-donor correlation (0.039) and included it as a blocking factor in the linear model. Since the analysis was run on different BeadChips, we corrected for this batch effect. Threshold for identification of DEGs was set with a false discovery rate (FDR) < 0.01 and a |log FC | ≥ 2. A broad gene list functional enrichment analysis for KEGG human pathways was performed using Enrichr [49]. All genes significantly regulated (FDR < 0.01) in day 1 calcium-macrophages compared to monocytes were used. For transcription factor target analysis, the TRRUST (Transcriptional Regulatory Relationships Unraveled by Sentence-based Text mining) database was used [28]. DEGs significantly up- or down-regulated (logFC ≥ 1.5, FDR < 0.01) on day 1 calcium-macrophages vs. monocytes and day 7 calcium-macrophages versus monocytes were inserted into the database (1d vs. monocytes: 494 total genes, 123 genes included in TRRUST; 7d vs monocytes: 501 total genes, 118 genes included in TRRUST). Microarray data were deposited in the NCBI GEO database under accession number GSE180027 and GSE180111.

### Proteomics

Protein lysates for proteomic analysis were prepared using RIPA lysis buffer. 1 ml lysis buffer was composed of 500 µl 2x RIPA buffer (2% Triton X100, 300 mM NaCl, 100 mM Tris-HCl pH 7.4, 1% Sodium deoxycholate, 0.2% SDS), 100 µl Complete™ Protease Inhibitor Cocktail (Merck), and 400 µl sterile distilled water. Cells were washed twice with cold 1X PBS, resuspended in lysis buffer, and incubated for 30 min on ice. Supernatants (lysates) containing proteins were collected after centrifugation at 10,000 × *g* for 15 min at 4 °C. The protein concentration in the lysates was determined using DC^TM^ Protein Assay (Bio Rad).

In total, 15 µg protein per sample were prepared for untargeted proteomics using a paramagnetic bead approach [53] and tandem mass tags (TMT) as described before [54] without sample acidification before reduction and alkylation. The TMT labeling (TMT-16-plex, Thermo Scientific, USA) was conducted with 89 µg label for 1 h at room temperature.

This protocol resulted in two fractions per sample, which were separated on a nano-UPLC system (Ultimate 3000, Dionex, USA) with trapping column (flow rate 5 µl/min, Acclaim PepMap 100 C18, 3 µm, nanoViper, 75 µm × 5 cm, Thermo Fisher, Germany) and analytical column (flow rate 0.3 µl/min, Acclaim PepMap 100 C18, 3 µm, nanoViper, 75 µm × 25 cm, Thermo Fisher, Germany) applying a non-linear gradient of 150 min. The separated peptides accessed the mass spectrometer (QExactive HF, Thermo Scientific, USA) via a chip-based ESI source (Nanomate, Advion, USA). The parameters were specified elsewhere as well [54], with the exception that not the top 10 but the top 15 most abundant precursor ions were isolated and fragmented. The obtained raw data have been deposited to the ProteomeXchange Consortium via the PRIDE [55] partner repository with the dataset identifier PXD026888.

MS raw data were processed against the UniprotKB reference proteome of *Homo sapiens* (3 May 2020) using ProteomeDiscoverer 2.4, selecting trypsin as cleavage reagent and allowing up to two missed cleavages. Oxidation of methionine and acetylation of the protein N-terminus were defined as variable modifications, while carbamidomethylation and TMT were selected as static modifications. Protein and peptide false discovery rates (FDRs) were set to 0.01. Only proteins for which at least two peptides were identified, one being unique, were kept. Reporter ion intensities were corrected using the factors provided by the manufacturer and normalized using the total peptide amount. These settings were also applied to identify bovine proteins, with the exception that the database search was performed against the UniprotKB reference proteome of *Homo sapiens* (3 May 2020) AND *Bos taurus* (8 December 2020). In contrast to the analysis of human proteins, for which the protein reporter intensities provided by ProteomeDiscoverer 2.4 were used, the effects on bovine proteins were investigated after filtering for peptides being unique for either *Homo sapiens* or *Bos taurus*, which were summarized to average reporter intensities for selected proteins before subsequent analyses.

All analyses and visualizations were performed in R-3.6.1 with the use of the packages mixOmics [56], limma [51], plyr [57], reshape2 [58], xlsx [59], DEP [60], calibrate [61], readxlbryan [62], qpcR [63], splitstackshape [64], tidyr [65], and Tmisc [66], ggplot2 [67], circlize [68], ggsci [69], ComplexHeatmap [70], dendsort [71], and dendextend [72].

With these packages, the obtained reporter intensities were normalized to pools containing a mixture of all analyzed samples, thus serving as internal control, and removing potential measurement bias. Next, the proteins were filtered for those being identified at least in triplicates, data were log2-transformed, and variance stabilized using the DEP package [60]. For selected comparisons, the average fold changes (Log2(FCs)) and p-values were calculated applying a Student’s t-test. The p-values were adjusted for multiple testing according to Benjamini & Hochberg, and proteins were considered significantly changed with adjusted p-value ≤ 0.05. A summary of obtained average Log2(FCs) and adjusted p-values for the here shown comparisons is stored in Supplementary Table 5.

### Western blot

Whole cell lysates from macrophages were prepared using RIPA lysis buffer after washing the cells with cold 1x PBS. To detect phosphorylation on proteins, the lysis buffer was supplemented with 1 mM of Sodium orthovanadate (Sigma), 50 mM Sodium fluoride (Sigma), and PhosSTOP ™ (Sigma). 30-35 µg of protein was loaded and resolved on a 12% SDS-PAGE gel and transferred to a polyvinylidene difluoride membrane (GE Healthcare) using a Bio Rad transfer apparatus according to the manufacturer’s protocols. Membranes were blocked using 5% milk powder (Saliter, Germany) dissolved in TBST (10 mM Tris, pH 8.0, 150 mM NaCl, 0.5% Tween 20) for 1 h. Membranes were incubated in primary antibody overnight at 4 °C followed by secondary antibody for 60 min. Blots were developed with the ECL system. Primary antibodies used were diluted in TBST buffer: Phospho-S6 Ribosomal Protein (Ser235/236) (2F9) Rabbit mAb 4856 - Cell Signaling (1:1000); phospho-p70 S6 Kinase (Thr389) Antibody 9205 Rabbit mAb—Cell Signaling (1:1000); COX2 (D5H5) XP® Rabbit mAb 12282- Cell Signaling (1:1000); and for housekeeping control: GAPDH (6C5) sc-32233 -mouse monoclonal IgG1- Santa Cruz (1:1000). Secondary antibodies Anti-rabbit IgG, HRP-linked Antibody 7074 - Cell Signaling (1:5000) and Anti-mouse IgG, HRP-linked Antibody 7076 - Cell Signaling (1:10000) were used.

The intensity of protein expression was measured using ImageJ 1.52 software and the ratio of intensity of the specific band to the intensity of the GAPDH band of the respective lane was calculated.

### THP-1 calcium-macrophage differentiation

In total, 4 × 10^5^/ml THP-1 (wildtype and CaSR-deficient) cells were differentiated with 20 ng/ml PMA for 24 h. Adherent cells were washed and calcium-macrophage differentiation was initiated with the addition of 0.85 mM CaCl_2_. After 48 h, cells were washed and cultured for additional 24 h. For some experiments, CaSR-deficient THP-1 cells were differentiated with 20 ng/ml PMA for 24 h. Adherent cells were washed and calcium-macrophage differentiation was initiated with the addition of 0.85 mM CaCl_2_ in the presence of 1 ng/ml PMA to facilitate macropinocytosis. After 48 h, cells were washed and cultured for additional 24 h. For inhibitor experiments, THP-1 cells were differentiated with 20 ng/ml PMA for 24 h. Adherent cells were washed and calcium-macrophage differentiation was initiated with the addition of 0.85 mM CaCl_2_ in the presence of MHY1485 (5 µM), S3I-20 (25 µM), or DMSO. No increased cell death was visually observed when the inhibitors were present, and cell death determination using trypan blue also revealed no increased cell death. THP-1 cells were imaged using Olympus IX50 inverted fluorescence phase contrast microscope (Olympus Life Sciences) and cellSens 1.4 software (Olympus Life Sciences).

### Scratch assay

Fresh isolated monocytes from donors were differentiated into macrophages for 7 days on IncuCyte® ImageLock 96-well Plates (Essen Bioscience, Sartorius) by seeding 1.5 × 10^5^ monocytes in 200 µl of differentiating media per well. Cells were washed with RPMI1640 + 10% FCS media and homogeneous, 700–800-micron wide scratch wounds were made in cell monolayers using Incucyte® WoundMaker (essenbioscience, Sartorius). Wells were washed to remove the detached cells and replenished with 200 µl of fresh media. Phase contrast, 10x magnification images in wide mode were taken every 2 h for 48 h with the Incucyte® live-cell analysis system. Incucyte® Analysis Software was used to calculate the Relative Wound Density (%), which is defined as the cell density in the wound area expressed relative to the cell density outside of the wound area over time.

### Cytokine and mediator measurements

Cytokines and mediators in cell supernatants were detected using ELISA according to the manufactor’s instructions. Kits used for various ELISA are as follows: Human IL-1β, IL-6, IL-10, IL-12p70, IL-18, TNF (all BD OptEIA); human IL-1a/IL-1F1, IL-23, IL-18bp, IL-1RA, osteopontin (all DuoSet ELISA, R&D); Prostaglandin E2 (Enzo Life Sciences), and thrombin-cleaved osteopontin (Immuno-Biological Laboratories).

### Fluorescence microscopy and nuclei quantification

Monocytes were differentiated into macrophages on Nunc™ Lab-Tek™ II Chamber Slide™ System (ThermoFischer) for 7 days. Cells were permeabilized with ice cold 100% methanol for 10 min at −20 °C and blocked and permeabilized for 60 min at room temperature with PBS, 5% FCS, and 0.3% Triton X-100. Cells were incubated overnight at 4 °C with primary antibody (1:200 β-Actin (D6A8) Rabbit mAB (Cell Signaling) in PBS / 1% BSA / 0.3% Triton X-100) followed goat anti-rabbit IgG Alex Fluor 488 (1:500, Life Technologies) and Hoechst 33342 (Invitrogen) for 1 h at room temperature. 10x images of multiple fields were taken using Microscope Axio Observer Z1 microscope (Carl ZEISS) from each donor. The percentage of multinucleated cells were calculated by counting (number of multinucleated cells in a field/ total number of cells in a field) *100.

### Cell elongation

For various experiments, cells were imaged using Olympus IX50 inverted fluorescence phasecontrast microscope (Olympus Life Sciences) and cellSens 1.4 software (Olympus Life Sciences). Cell length and width of all adherent cells within the image were determined using ImageJ (version 2.1.0/1.53c). Cell elongation was calculated by dividing the cell length by cell width.

### Calciprotein particle (CPP) preparation and stimulation

To prepare calciprotein particles, 5 µl of 100 mM CaCl_2_ was added to 200 µl of room temperature RPMI1640 + 10% FCS, and centrifuged at 16,000 × *g* for 2 h at 21 °C. When required, 15 µM of Calcein (Sigma) was added to obtain calcein-stained CPPs. CPPs acquired from 200 µl of CPP-media is considered as 1xCPPs. To stimulate cells with 1xCPPs, the pelleted CPPs were resuspended with customized RPMI (1 mM phosphate) + 10% FCS to avoid further spontaneous CPP formation.

### Macropinocytosis assays

Macropinocytosis was detected by quantifying the uptake of Calcein-stained CPPs or DQ-BSA red by the cells. Freshly isolated monocytes were incubated at 37 °C with 5% CO_2_ with 1x Calcein-CPPs in 1 mM phosphate RPMI + 10% FCS with and without 2.5 mM CaCl_2_ and 10 µg/ml DQ-BSA red (Invitrogen, D12051) for 4 h. To detect CPP and lysosome colocalization, 5 nM LysoTracker™ Deep Red (Invitrogen, L12492) was added into the wells 20 min before the end of the time point. Cells were collected into 1.5 ml Eppendorf tubes, washed with PBS, and resuspended in 30 µl of PBS to measure with Amnis® ImageStreamX Mark II Imaging Flow Cytometer (INSPIRE for the ISX mkII Version 200.1.388.0). Uptake of Calcein-CPPs or DQ-BSA red was quantified using Amins IDEAS version 6.2 software. The gating strategy is visualized in Supplementary Fig. 13. To quantify colocalization, the colocalization wizard was utilized.

### Intracellular staining

Monocytes were stimulated in RPMI1640 + 10% FCS with 2.5 mM CaCl_2_ for 3 or 16 h. For some experiments, monocytes were incubated in the presence of mTOR activator MHY1485 (MedChemExpress, HY-B0795) and STAT3 inhibitor S3I-201 (Sigma-Aldrich, 573102). Cells were washed and fixed with 3.7% Paraformaldehyde for 20 min. Cells were stained with the primary antibodies for TFEB (1:100, Invitrogen, PA1-31552), LAMP2-FITC (clone H4B4, Miltenyi), or STAT3-APC (clone M59-50, BD Biosciences) for 30 min in permeabilization buffer (0.1% Triton-X in PBS with 10% AB serum). Secondary staining for TFEB was done by incubating cells with fluorescence conjugated secondary antibody donkey anti-goat IgG Alexa Fluor 647 (for TFEB, 1:500, Invitrogen) and Hoechst 33342 (Invitrogen) for 30 min in permeabilization buffer. Staining was detected using Amnis® ImageStream^X^ Mark II Imaging Flow Cytometer (INSPIRE for the ISX mkII Version 200.1.388.0) by collecting 5000–10,000 cells from each sample. The gating strategy to detect LAMP2 and lysotracker fluorescence is shown in Supplementary Fig. 14. Dead monocytes were excluded according to their lesser or absent LAMP2 or Lysotracker staining which matched the visual impression obtained with the respective brightfield image. However, there was no difference in cell death with and without calcium after 3 h of stimulation. Nuclear translocation was quantified using the Nuclear Localization wizard of the Amnis IDEAS version 6.2 software (gating strategy for transcription factors is visualized in Supplementary Fig. 15). In the nuclear translocation experiments, viable monocytes were identified according to their higher nuclear staining with Hoechst 33342 and intact nuclear structure compared to dead monocytes. When MHY1485 or S3I-201 were used, cell death was also quantified using trypan blue staining and microscopic evaluation. Both substances did not induce relevant cell death during the 3 h incubation period, >90% of monocytes were viable. For STAT3 translocation, the staining was standardized using IL-6 as a positive control. The results for 2 donors (60 min stimulation of monocytes with 10 ng/ml IL-6) and representative images are shown in Supplementary Fig. 16.

### LysoSensor and CellProfiler

The LysoSensor dye was used to assess lysosomal pH in differentiated THP-1 cells. For differentiation, 5 × 10^5^ CaSR wildtype and CaSR-deficient cells were seeded in 1 ml RPMI 1640 supplemented with 10% FCS containing 20 ng/ml PMA in a 24-well plate and incubated for 48 h at 37 °C and 5% CO_2_. Medium was removed and cells were washed with PBS. 200 µl Accutase® was added and cells were incubated for 30 min at 37 °C. Detached cells were harvested and washed in PBS. 5 × 10^5^ cells were seeded in 400 µl RPMI 1640 with 10% FCS into a 48-well plate coated with 1.5% agarose and stimulated with 1 mM CaCl_2_ for 3 h. 15 min prior to the end of incubation, 1 µM LysoSensor™ Green DND-189 (Invitrogen) was added per well. Cells were washed two times with PBS and LysoSensor intensity was measured using Amnis® ImageStreamXMark II analysis (Ch04-Brightfield, Ch02-LysoSensor, INSPIRE for the ISX mkII version 200.1.388.0) (gating strategy shown in Supplementary Fig. 17). Dead THP-1 cells were identified and excluded according to their loss of LysoSensor staining which matched the visual impression obtained with the brightfield image.

Images obtained with Amnis® ImageStreamXMark II were bulk exported (12 × 14 cells, 168 cells per condition), and analyzed with CellProfiler (version 4.1.3) [73]. Lysosomes were enhanced with the “EnhanceOrSuppressFeatures” module, and subsequently identified using the “IdentifyPrimaryObjects” module. The intensity of the identified lysosomes were measured in the original image using the “MeasureObjectIntensity”. The mean intensity for every identified object (lysosomes) was used for further analysis.

### Proliferation assay

Freshly isolated monocytes were stained with 4.5 µM carboxyfluorescein diacetate succinimidyl ester (CFSE) for 10 min, washed, and then differentiated into calcium-macrophages, GM-CSF-macrophages, and M-CSF-macrophages by incubating 5 × 10^5^/ml monocytes with respective differentiation media for 7 days at 37 °C with 5% CO_2_. Cell death was evaluated using propidium iodide on day 1, day 2, and day 7. CFSE staining was measured on day 7 using the BD LSRFortessa Flow Cytometer (BD Biosciences). Data were analyzed using FlowJo 10 (BD Biosciences) (gating strategy shown in Supplementary Fig. 18).

### Graphs and statistics

Graphs and statistics were prepared with GraphPad Prism 8.4.3. Bar charts represent mean + s.e.m. and individual values of each experiment are represented as symbols in bars. Normal distribution of data was checked using the Shapiro-Wilk test. Statistical significance was determined accordingly using the two-tailed non-parametric, unpaired Mann-Whitney U tests or *t* test, confidence interval of 95%. The sample size was between 3 and 10 independent experiments with monocytes/macrophages from different human donors. Based on previous experiments, a strong cytokine or functional response was expected using human monocytes/macrophages, therefore the obtained statistical results were robust. No samples were excluded from the analysis.

## Supplementary information

Supplementary Data

Checklist

Supplementary Table 1

Supplementary Table 5

Supplementary Video 1

## Data Availability

The datasets generated during and/or analyzed during the current study are available from the corresponding author on reasonable request. Public datasets: Microarray data: NCBI GEO GSE180027 and GSE180111, scRNA-seq data: NCBI GEO GSE180113, Proteomics data: PRIDE PXD026888.
