# Supplementary material for: Danger signal extracellular calcium initiates differentiation of monocytes into SPP1/osteopontin-producing macrophages
Source: Cell Death Dis. 2022 Jan 12;13(1):53. doi: 10.1038/s41419-022-04507-3 (PMC8755842; doi:10.1038/s41419-022-04507-3)
Supplement: Supplementary file 1 — Supplementary Data [file 41419_2022_4507_MOESM1_ESM.docx]

**Supplementary Figures & Methods**


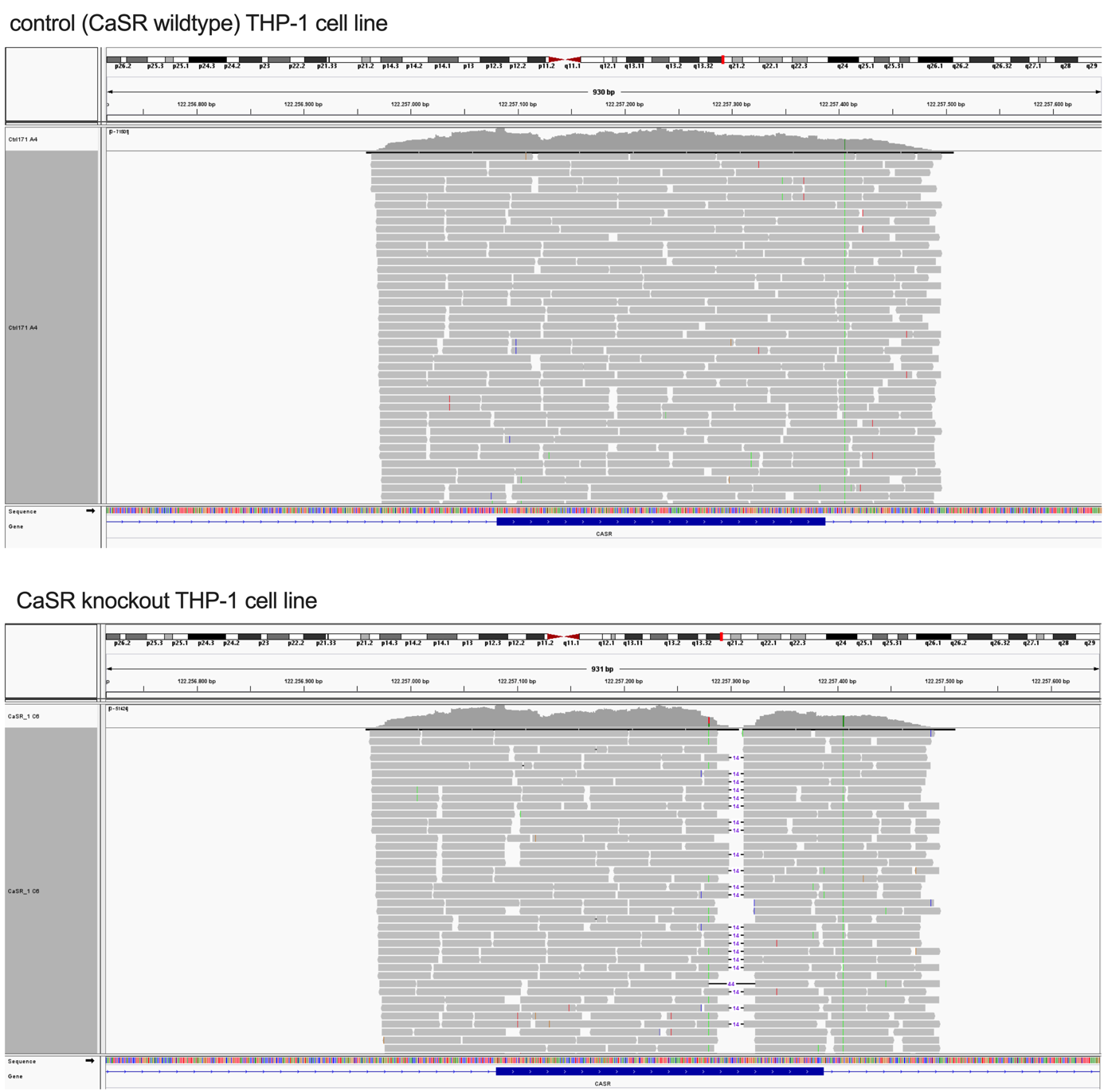


Supplementary Figure 1

The sequencing reads and coverage for clone CaSR_1 C6 (CaSR knockout) compared to wildtype (Ctrl171 A4). Sequencing (n=1) reveals most alleles contain deletions of 14 or 44 bp at the edited sites.


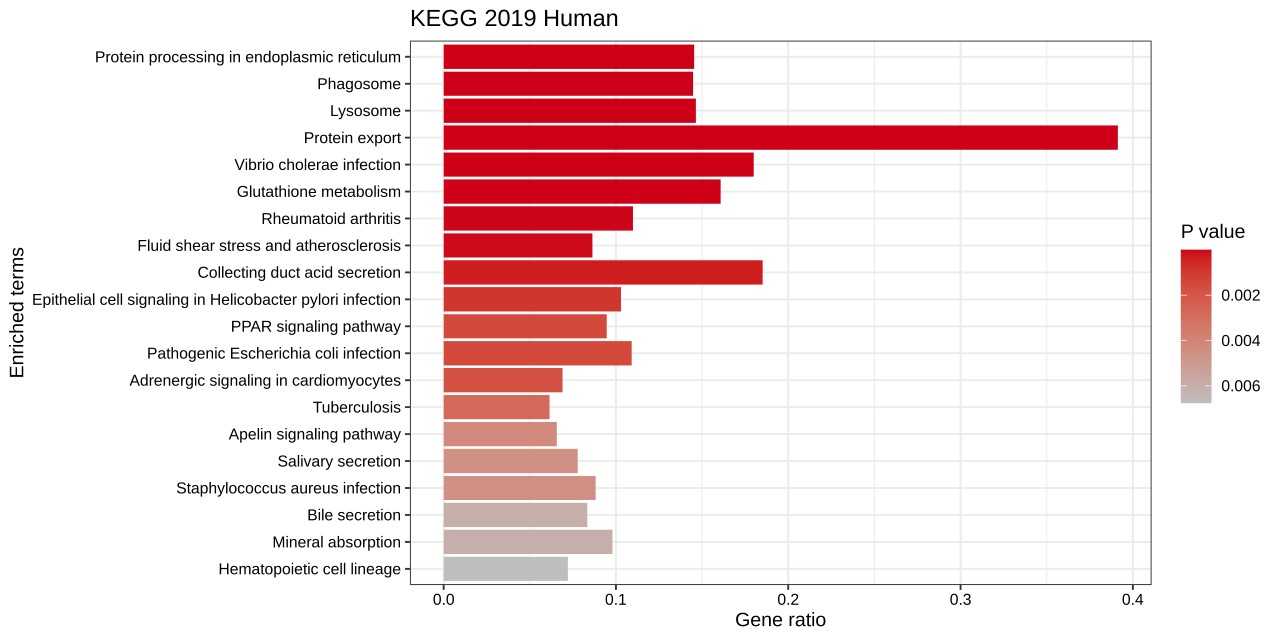


Supplementary Figure 2:

KEGG pathway analysis of HashTag3-labelled calcium-macrophages: The 20 most significantly enriched pathways in KEGG according to enrichR with gene ratio being the share of all genes in the respective pathway that are detected in the set of DEGs in calcium-macrophages while the coloring indicates p-values.


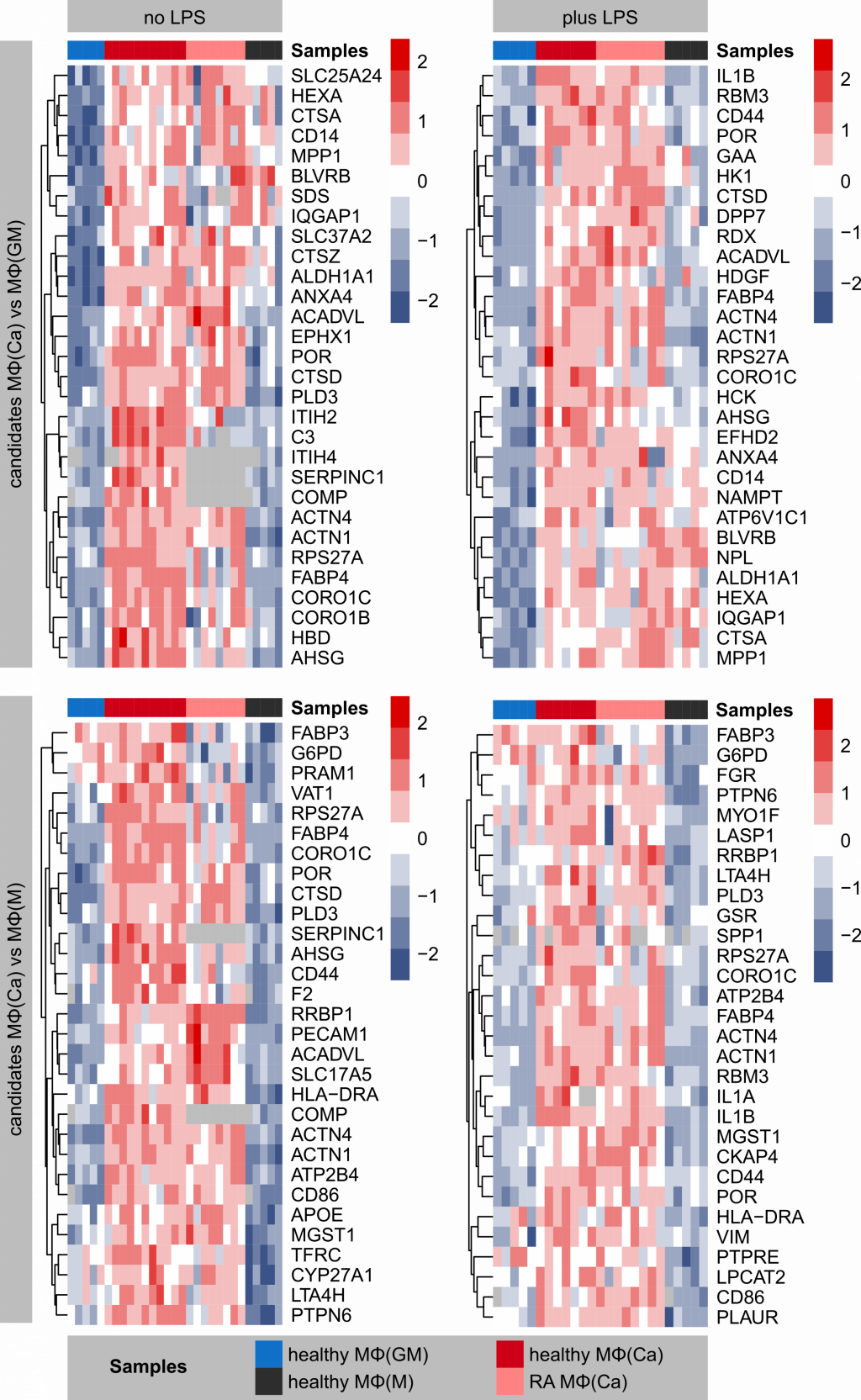


Supplementary Figure 3: Proteomic analysis of cell lysates of calcium‑macrophages (Ca, healthy n=11, RA n=8), GM‑CSF‑macrophages (GM, n=5), and M‑CSF‑macrophages (M, n=5) differentiated for 7 days. Shown are top30 proteins significantly regulated in resting (no LPS) or LPS-stimulated (plus LPS) calcium‑macrophages compared to GM‑CSF‑macrophages or M-CSF-macrophages.


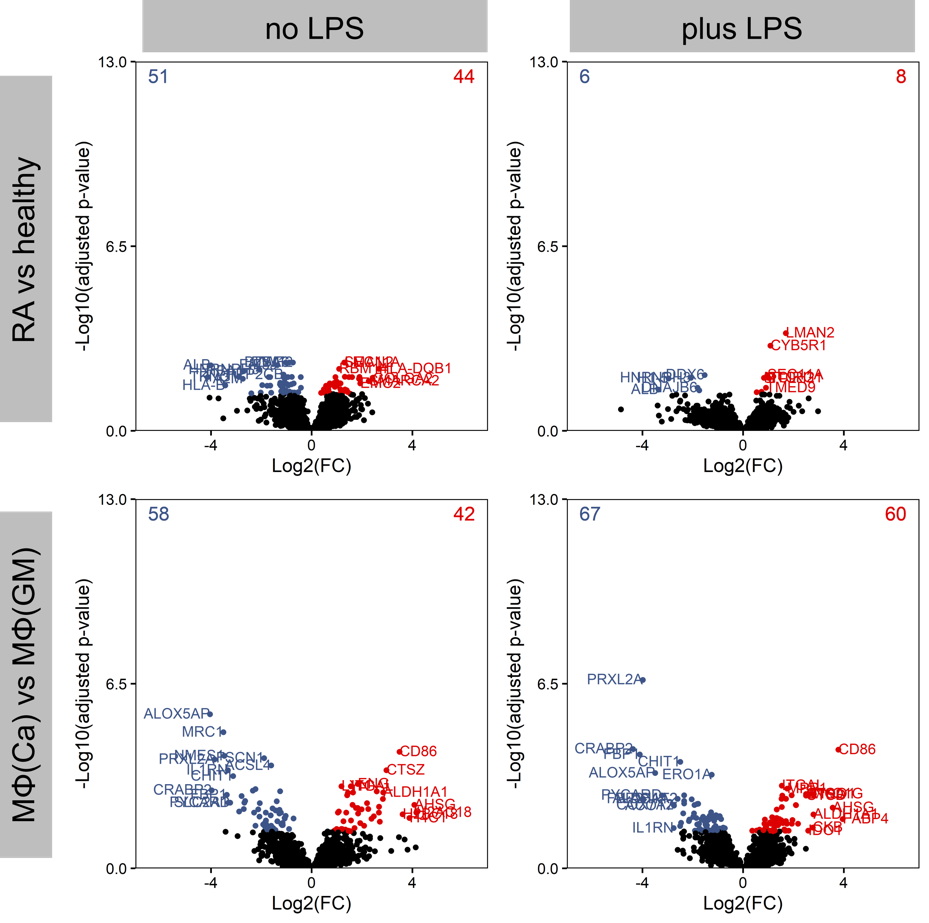


Supplementary Figure 4:

Log2(FCs) and -Log10(adjusted p-values) of proteins for the comparisons RA MΦ (Ca) vs healthy MΦ (Ca) and RA MΦ (Ca) vs RA MΦ (GM) with and without LPS stimulation. Significantly (adjusted p-value ≤0.05) increased (red) and decreased (blue) proteins are labeled and numbers of significantly affected proteins are given in the corners.


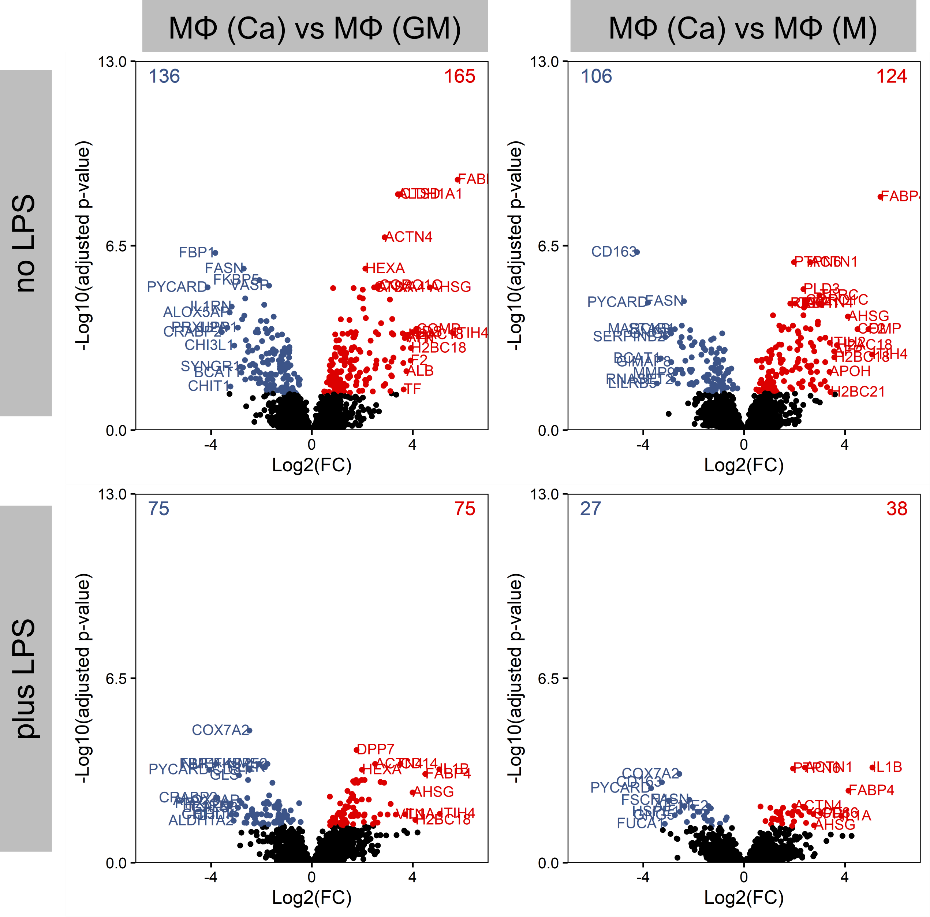


Supplementary Figure 5:

Log2(FCs) and -Log10(adjusted p-values) of proteins for the comparisons MΦ (Ca) vs MΦ (GM) and MΦ (Ca) vs MΦ (M) with and without LPS stimulation. Significantly (adjusted p-value ≤0.05) increased (red) and decreased (blue) proteins are labeled and numbers of significantly affected proteins are given in the corners.


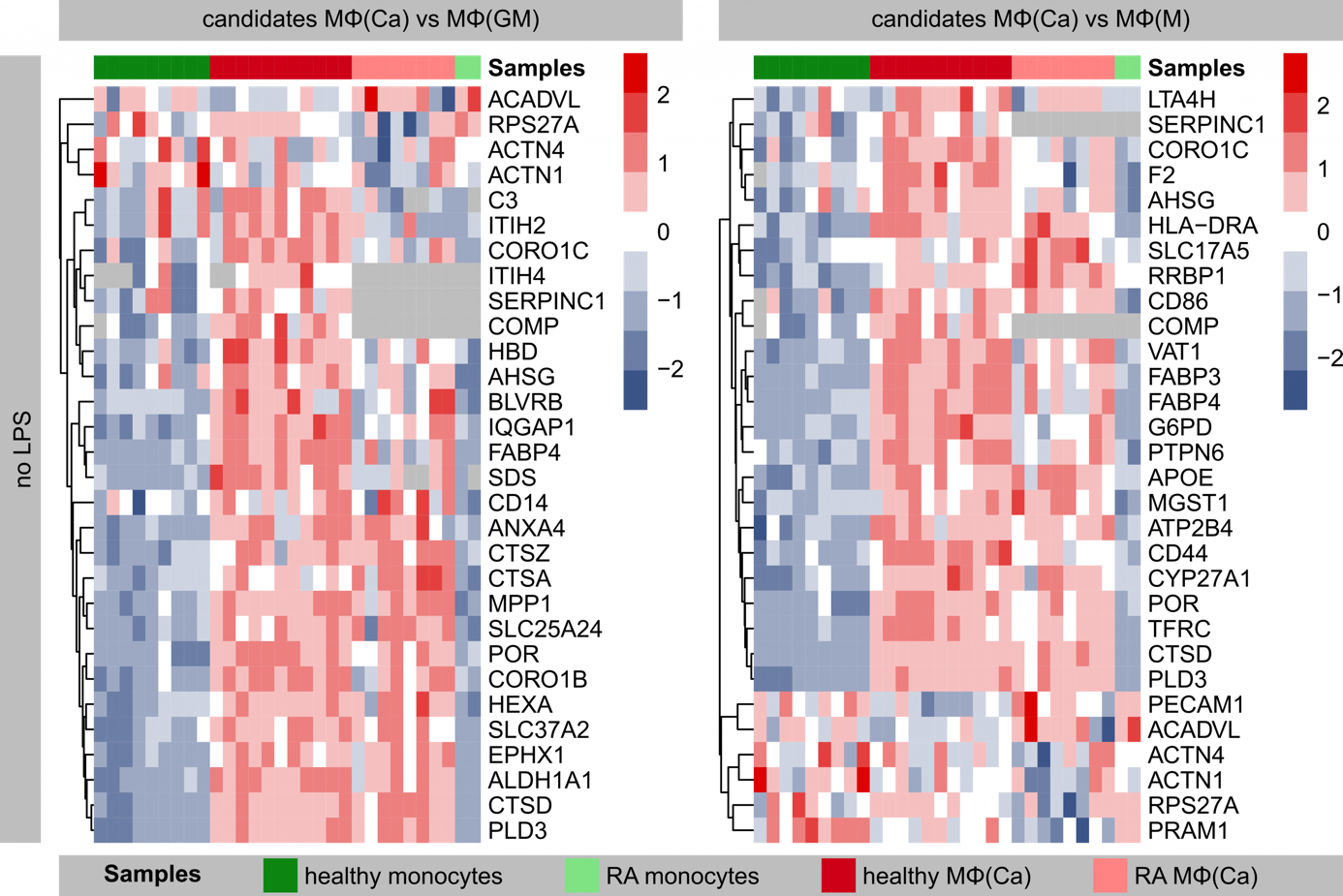


Supplementary Figure 6: Proteomic analysis of cell lysates of calcium‑macrophages (Ca, healthy n=11, RA n=8) differentiated for 7 days, and freshly isolated monocytes (healthy n=9, RA n=2). Shown are top30 proteins significantly regulated in resting (no LPS) calcium‑macrophages compared to GM‑CSF‑macrophages or M-CSF-macrophages (candidate proteins identified in Supplementary figure 3.


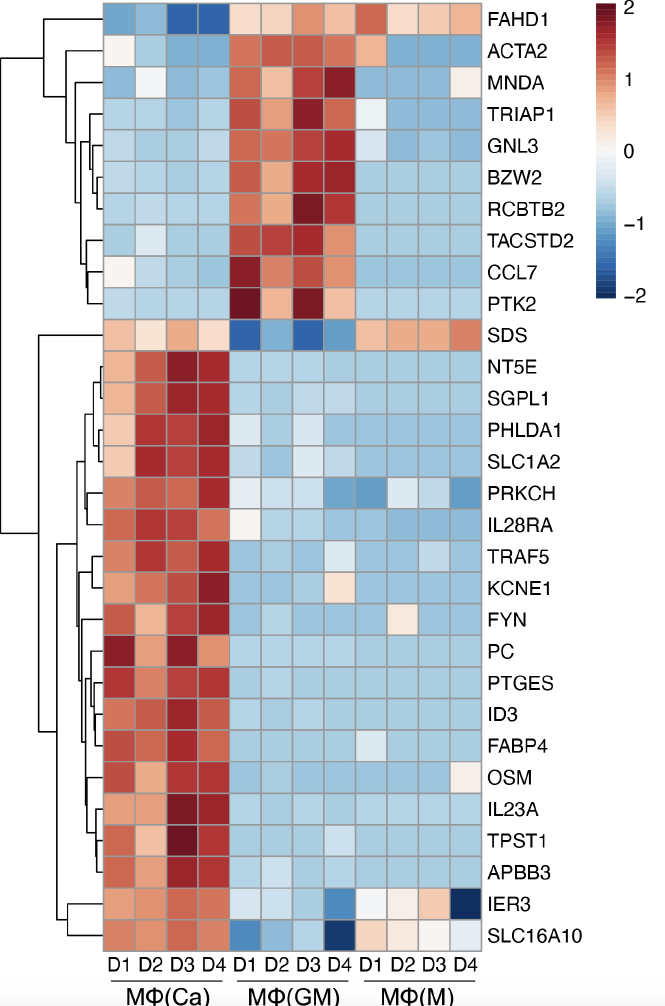


Supplementary Figure 7:

Top 30 genes significantly regulated in LPS-stimulated calcium-macrophages compared to GM-CSF-macrophages. Macrophages were differentiated from monocytes of four different donors (D1-D4) for seven days, stimulated with 10 ng/ml LPS for 24 hours, and gene expression was analyzed using DNA microarray.


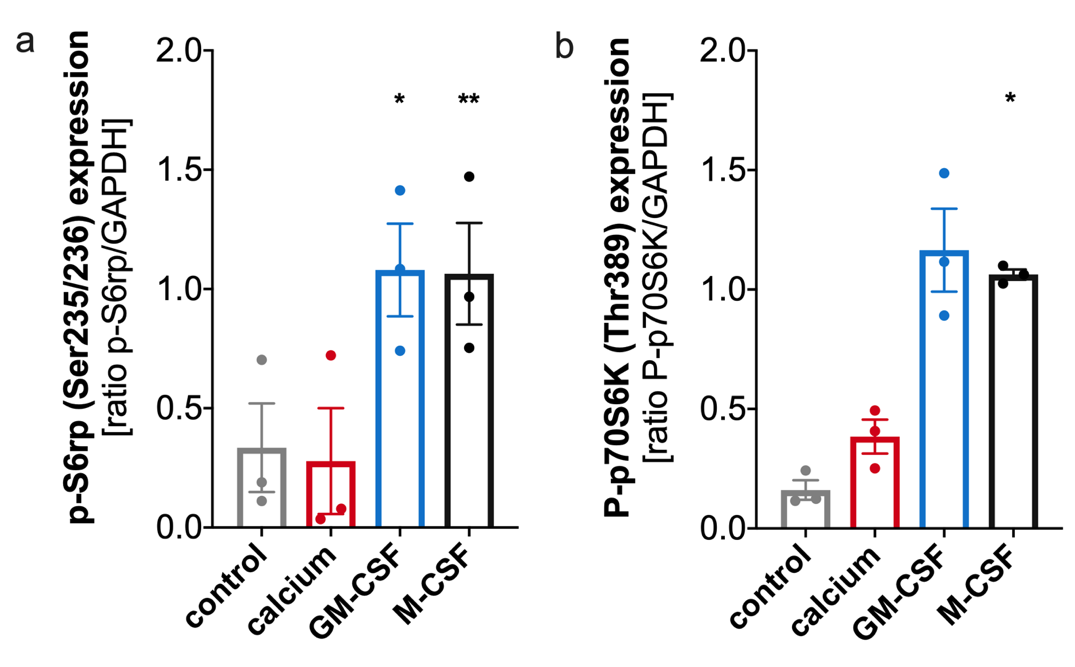


Supplementary Figure 8: Ratio of expression of phosphorylated S6rp (P‑S6rp) and p70S6K (P‑p70S6K) to the respective GAPDH loading controls in monocytes differentiated for 1 day in the presence of calcium, GM-CSF, or M-CSF (n=3). Bar charts show mean ± s.e.m. Statistical analysis was performed using paired two‑tailed t‑test (p‑value compared to calcium * ≤0.05, ** ≤0.01).


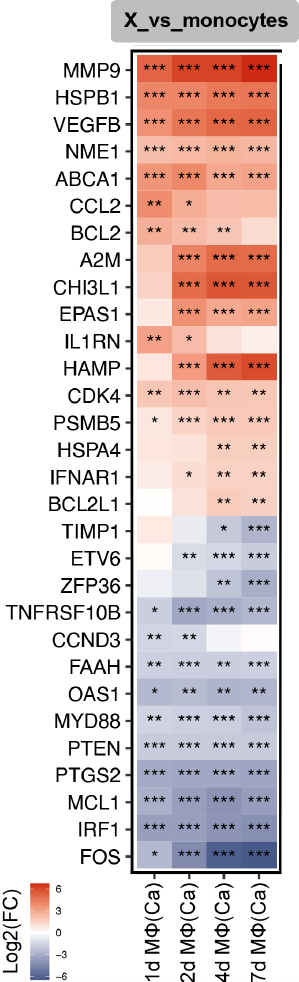


Supplementary Figure 9: STAT3 target genes (identified with the TRRUST database) significantly regulated in day 1, 2, 4 or 7 calcium‑macrophages compared to monocytes. Calcium‑macrophages were differentiated from monocytes of four different donors for the indicated times, and gene expression was analyzed using DNA microarray. Significant changes are indicated with asterisks (adjusted p value * ≤0.05, ** ≤0.01, *** ≤0.001).


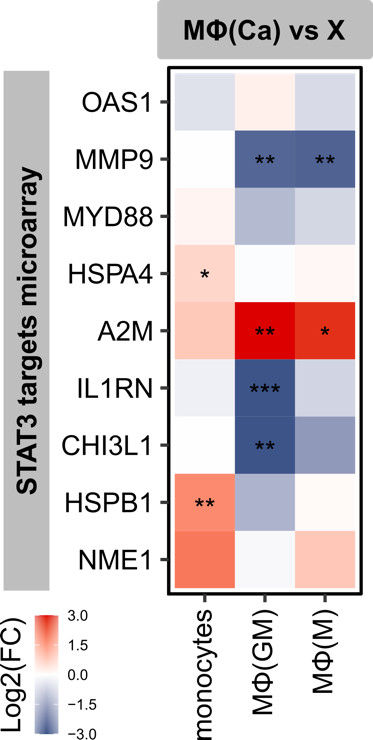


Supplementary Figure 10: STAT3 target genes (identified for the microarray data with the TRRUST database) regulated on the proteome level in calcium‑macrophages compared to monocytes, GM-CSF-macrophages, or M-CSF-macrophages. Macrophages were differentiated from monocytes of 7 different donors (calcium-macrophages) or 5 different donors (GM-CSF- and M-CSF-macrophages) for seven days. Significant changes are indicated with asterisks (adjusted p value * ≤0.05, ** ≤0.01, *** ≤0.001).


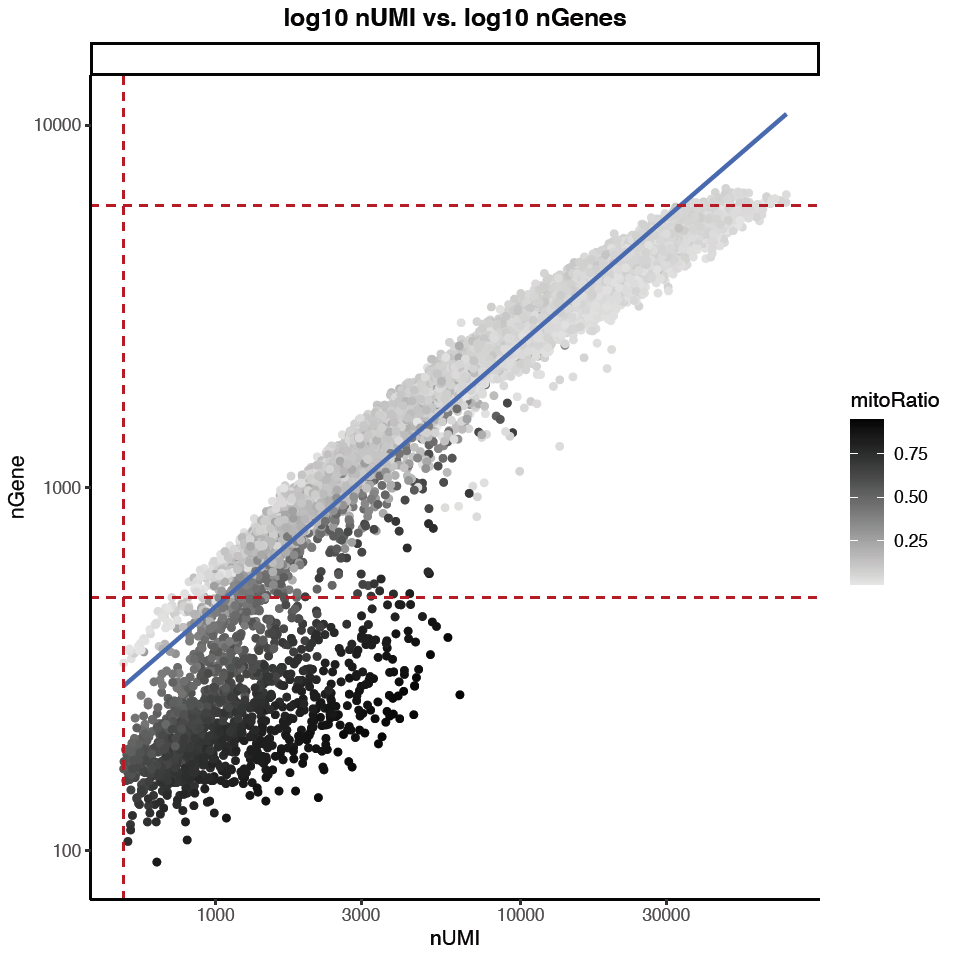


Supplementary Figure 11:

Number of genes (nGenes) plotted against the number of UMI (nUMI).


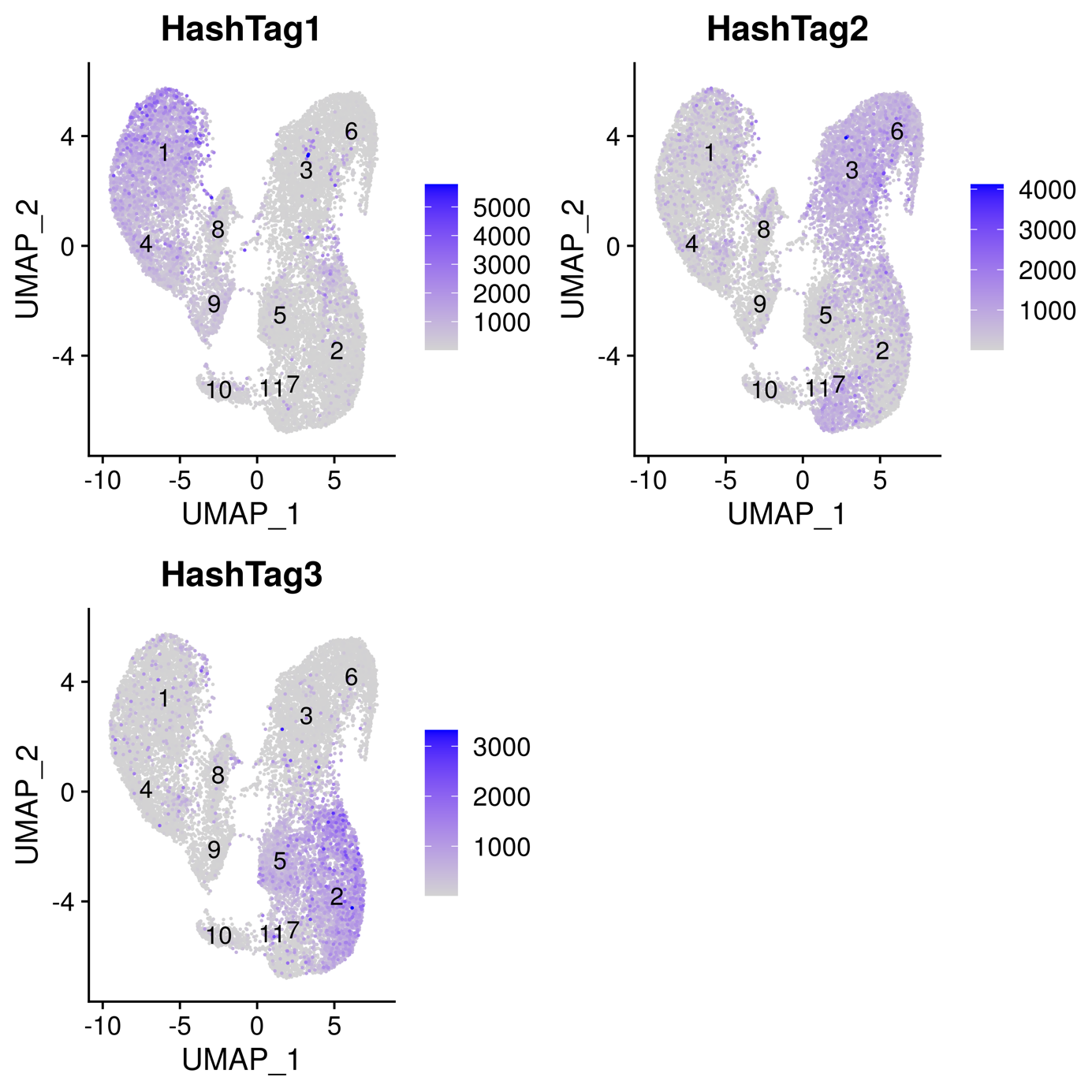


Supplementary Figure 12:

UMAP and dot plots of the distribution of oligo-tagged antibodies (HashTag1 = GM-CSF-macrophages, HashTag2 = M-CSF-macrophages, HashTag3 = calcium-macrophages).


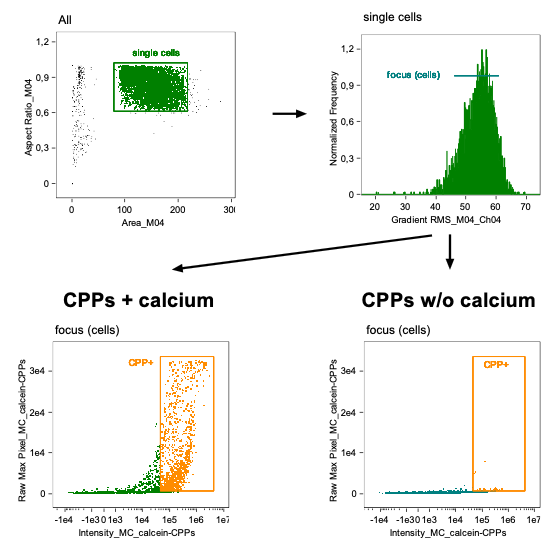


Supplementary Figure 13:

Gating strategy for detection of calcein-CPP uptake in monocytes using Amnis® ImageStream^X^ Mark II Imaging Flow Cytometer.


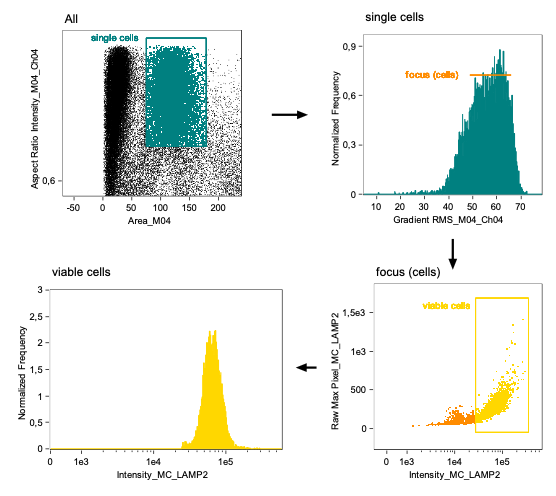


Supplementary Figure 14:

Gating strategy for detection of LAMP2 or lysotracker fluorescence in monocytes using Amnis® ImageStream^X^ Mark II Imaging Flow Cytometer.


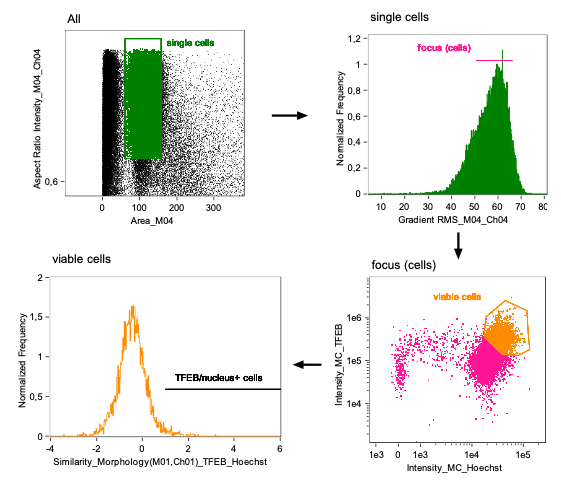


Supplementary Figure 15:

Gating strategy for detection of transcription factor (TFEB, STAT3) nuclear translocation in monocytes using Amnis® ImageStream^X^ Mark II Imaging Flow Cytometer.


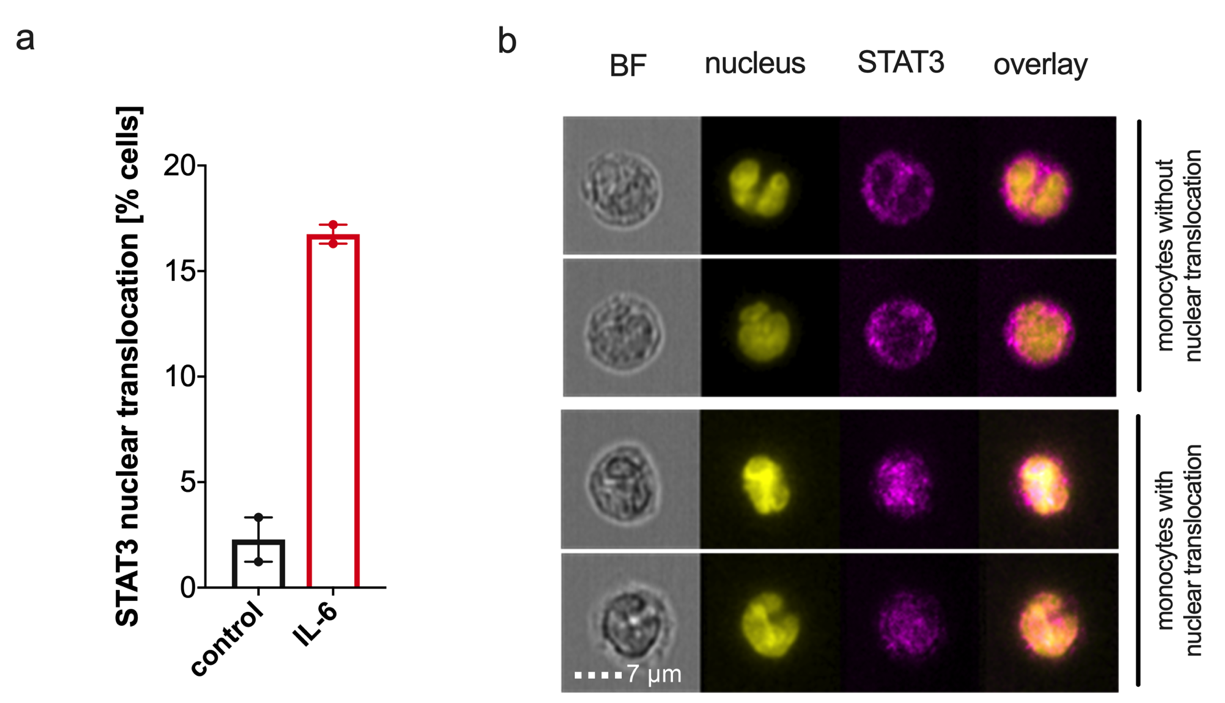


Supplementary Figure 16:

IL-6‑dependent nuclear translocation of STAT3 in monocytes stimulated with and without 10ng/ml IL-6 for 60 minutes. Analysis was performed using ImageStreamX Mark II and 5,000‑10,000 monocytes were imaged from 2 different donors. Representative images showing brightfield (BF), nucleus, STAT3, and an overlay of nucleus and STAT3 staining (b). Bar charts show mean ± s.e.m. (a).


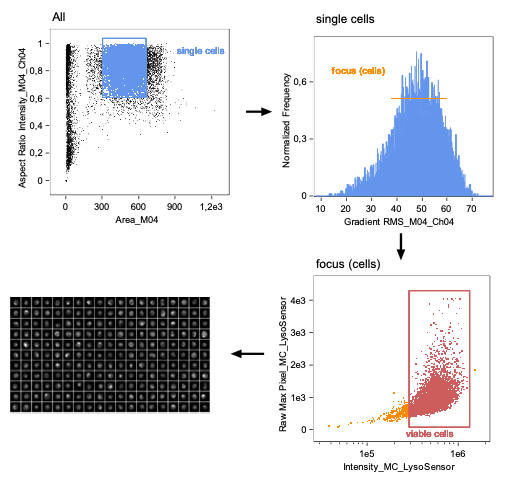


Supplementary Figure 17:

Gating strategy for detection of LysoSensor fluorescence in monocytes using Amnis® ImageStream^X^ Mark II Imaging Flow Cytometer.

Supplementary Figure 18:

Gating strategy for detection of CFSE fluorescence in monocytes/macrophages using flow cytometry.

| **Index** | **Name** | **Overlap** | **p-value** | **Adjusted**  **p-value** | **Odds Ratio** | **Score** |
| --- | --- | --- | --- | --- | --- | --- |
| 1 | Rheumatoid arthritis | 30/91 | 3.22E-09 | 9.57E-07 | 4.36 | 85.26 |
| 2 | Fluid shear stress and atherosclerosis | 38/139 | 1.06E-08 | 1.58E-06 | 3.34 | 61.35 |
| 3 | Lysosome | 34/123 | 4.61E-08 | 4.57E-06 | 3.39 | 57.23 |
| 4 | Vibrio cholerae infection | 19/50 | 1.93E-07 | 1.43E-05 | 5.41 | 83.70 |
| 5 | Antigen processing and presentation | 24/77 | 3.86E-07 | 2.30E-05 | 4.00 | 59.13 |
| 6 | Human cytomegalovirus infection | 48/225 | 6.36E-07 | 3.15E-05 | 2.41 | 34.39 |
| 7 | Phagosome | 35/152 | 3.37E-06 | 1.43E-04 | 2.65 | 33.39 |
| 8 | Protein export | 11/23 | 5.31E-06 | 1.97E-04 | 8.07 | 98.06 |
| 9 | Epithelial cell signaling in Helicobacter pylori infection | 20/68 | 9.49E-06 | 3.11E-04 | 3.68 | 42.54 |
| 10 | Hematopoietic cell lineage | 25/97 | 1.05E-05 | 3.11E-04 | 3.07 | 35.19 |
| 11 | Protein processing in ER | 35/165 | 2.27E-05 | 6.13E-04 | 2.38 | 25.49 |
| 12 | Estrogen signaling pathway | 30/137 | 4.55E-05 | 0.001125037 | 2.48 | 24.79 |
| 13 | Proteasome | 14/45 | 1.04E-04 | 0.002368641 | 3.98 | 36.50 |
| 14 | Neurotrophin signaling pathway | 26/119 | 1.48E-04 | 0.003131108 | 2.47 | 21.78 |
| 15 | Oxidative phosphorylation | 28/133 | 1.64E-04 | 0.003252792 | 2.36 | 20.53 |
| 16 | Collecting duct acid secretion | 10/27 | 2.03E-04 | 0.003551813 | 5.18 | 44.01 |
| 17 | Chemokine signaling pathway | 36/190 | 2.03E-04 | 0.003551813 | 2.07 | 17.58 |
| 18 | Legionellosis | 15/55 | 3.05E-04 | 0.005032738 | 3.30 | 26.75 |
| 19 | Pathways in cancer | 79/530 | 4.12E-04 | 0.00630445 | 1.56 | 12.14 |
| 20 | T-cell leukemia virus 1 infection | 39/219 | 4.25E-04 | 0.00630445 | 1.92 | 14.88 |
| 21 | Human papillomavirus infection | 53/330 | 6.36E-04 | 0.008991562 | 1.70 | 12.48 |
| 22 | mTOR signaling pathway | 29/152 | 7.17E-04 | 0.009685515 | 2.08 | 15.07 |
| Supplementary Table 2: KEGG pathway analysis of genes significantly regulated (p<0.01) in day 1 calcium-macrophages compared to monocytes (n=4). The analysis was performed with the online tool Enrichr. | | | | | | |

| **#** | **Key TF** | **Description** | **overlapping genes** | **P value** | **FDR** |
| --- | --- | --- | --- | --- | --- |
| Day 1 calcium-macrophages versus monocytes | | | | | |
| 1 | NFKB1 | nuclear factor of kappa light polypeptide gene enhancer in B-cells 1 | 27 | 2.96e-23 | 3.34e-21 |
| 2 | RELA | v-rel reticuloendotheliosis viral oncogene homolog A (avian) | 24 | 1.42e-19 | 8.02e-18 |
| 3 | SP1 | Sp1 transcription factor | 27 | 3.1e-18 | 1.17e-16 |
| 4 | JUN | jun proto-oncogene | 17 | 1.01e-16 | 2.86e-15 |
| 5 | STAT3 | signal transducer and activator of transcription 3 (acute-phase response factor) | 16 | 1.04e-15 | 2.35e-14 |
| 6 | TP53 | tumor protein p53 | 16 | 1.05e-14 | 1.97e-13 |
| 7 | ETS1 | v-ets erythroblastosis virus E26 oncogene homolog 1 (avian) | 9 | 2.2e-09 | 3.56e-08 |
| 8 | SP3 | Sp3 transcription factor | 10 | 3.35e-09 | 4.73e-08 |
| 9 | STAT1 | signal transducer and activator of transcription 1, 91kDa | 9 | 3.83e-09 | 4.81e-08 |
| 10 | EGR1 | early growth response 1 | 9 | 5.82e-09 | 6.58e-08 |
| Day 7 calcium-macrophages versus monocytes | | | | | |
| 1 | SP1 | Sp1 transcription factor | 47 | 2.77e-31 | 3.72e-29 |
| 2 | NFKB1 | nuclear factor of kappa light polypeptide gene enhancer in B-cells 1 | 36 | 1.24e-26 | 8.33e-25 |
| 3 | RELA | v-rel reticuloendotheliosis viral oncogene homolog A (avian) | 34 | 1.93e-24 | 7.66e-23 |
| 4 | STAT3 | signal transducer and activator of transcription 3 (acute-phase response factor) | 26 | 2.29e-24 | 7.66e-23 |
| 5 | TP53 | tumor protein p53 | 23 | 6.27e-19 | 1.68e-17 |
| 6 | SPI1 | spleen focus forming virus (SFFV) proviral integration oncogene spi1 | 16 | 5.72e-18 | 1.28e-16 |
| 7 | JUN | jun proto-oncogene | 19 | 4.84e-15 | 9.26e-14 |
| 8 | EGR1 | early growth response 1 | 14 | 9.47e-13 | 1.59e-11 |
| 9 | STAT1 | signal transducer and activator of transcription 1, 91kDa | 13 | 9.06e-12 | 1.35e-10 |
| 10 | MYC | v-myc myelocytomatosis viral oncogene homolog (avian) | 12 | 1.22e-09 | 1.64e-08 |
| Supplementary Table 3: Transcription factor (TF) target gene interaction of genes significantly regulated (p<0.01, logFC>1.5) in day 1 calcium-macrophages compared to monocytes and day 7 calcium-macrophages compared to monocytes (n=4). The analysis was performed with the online tool TRRUST. | | | | | |

| **Supplementary Table 4:**  **Characteristics of patients with rheumatoid arthritis** | |
| --- | --- |
| **Characteristics** | **Value** |
| Number of patients (female/male) | 30 (17/13) |
| Age (years) [median (range)] | 60 (32-80) |
| Age at onset (years) [median (range)] | 58 (30-79) |
| Disease duration (years) [median (range)] | 0.5 (0-31) |
| Patients positive for rheumatoid factor IgM | 29 |
| Patients positive for anti-CCP Abs | 29 |
| Therapy |  |
| Without DMARD | 12 |
| Methotrexate | 15 |
| Sulfasalazine | 2 |
| Baricitinib | 1 |
